# Chronic inflammation in post-acute sequelae of COVID-19 modulates gut microbiome: a review of literature on COVID-19 sequelae and gut dysbiosis

**DOI:** 10.1186/s10020-024-00986-6

**Published:** 2025-01-23

**Authors:** Najeeha Talat Iqbal, Hana Khan, Aqsa Khalid, Syed Faisal Mahmood, Nosheen Nasir, Iffat Khanum, Isadora de Siqueira, Wes Van Voorhis

**Affiliations:** 1 Department of Biological and Biomedical Sciences, Department of Pediatrics and Child Health, Aga Khan University, Stadium Road, P. O Box 3500, Karachi, 74800 Pakistan; 2 Undergraduate Medical Education (UGME), Year II, Aga Khan University, Karachi, Pakistan; 3 Department of Pediatrics & Child Health, Aga Khan University, Karachi, Pakistan; 4 Department of Medicine, Aga Khan University, Karachi, Pakistan; 5 Fiocruz Salvador, Instituto Gonçalo Moniz, Salvador, BA Brazil; 6 Center for Emerging and Re-emerging Infectious Diseases (CERID), University of Washington, Seattle, USA

**Keywords:** Microbiota, COVID-19, Gut-brain axis, Inflammation, Long COVID, PASC

## Abstract

**Background:**

Long COVID or Post-acute sequelae of COVID-19 is an emerging syndrome, recognized in COVID-19 patients who suffer from mild to severe illness and do not recover completely. Most studies define Long COVID, through symptoms like fatigue, brain fog, joint pain, and headache prevailing four or more weeks post-initial infection. Global variations in Long COVID presentation and symptoms make it challenging to standardize features of Long COVID. Long COVID appears to be accompanied by an auto-immune multi-faceted syndrome where the virus or viral antigen persistence causes continuous stimulation of the immune response, resulting in multi-organ immune dysregulation.

**Main text:**

This review is focused on understanding the risk factors of Long COVID with a special emphasis on the dysregulation of the gut-brain axis. Two proposed mechanisms are discussed here. The first mechanism is related to the dysfunction of angiotensin-converting enzyme 2 receptor due to Severe Acute Respiratory Syndrome Corona Virus 2 infection, leading to impaired mTOR pathway activation, reduced AMP secretion, and causing dysbiotic changes in the gut. Secondly, gut-brain axis dysregulation accompanied by decreased production of short-chain fatty acids, impaired enteroendocrine cell function, and increased leakiness of the gut, which favors translocation of pathogens or lipopolysaccharide in circulation causing the release of pro-inflammatory cytokines. The altered Hypothalamic-Pituitary-Adrenal axis is accompanied by the reduced level of neurotransmitter, and decreased stimulation of the vagus nerve, which may cause neuroinflammation and dysregulation of serum cortisol levels. The dysbiotic microbiome in Long COVID patients is characterized by a decrease in beneficial short chain fatty acid-producing bacteria (Faecalibacterium, Ruminococcus, Dorea, and Bifidobacterium) and an increase in opportunistic bacteria (Corynebacterium, Streptococcus, Enterococcus). This dysbiosis is transient and may be impacted by interventions including probiotics, and dietary supplements.

**Conclusions:**

Further studies are required to understand the geographic variation, racial and ethnic differences in phenotypes of Long COVID, the influence of viral strains on existing and emerging phenotypes, to explore long-term effects of gut dysbiosis, and gut-brain axis dysregulation, as well as the potential role of diet and probiotics in alleviating those symptoms.

## Background

SARS-CoV-2 or COVID-19 has emerged as a global health concern due to unusually high morbidity and mortality rates (Markov et al. 2023), and therefore gained attention from the scientific community for debate and discussion (Dashraath et al. 2020). Although COVID-19 symptoms last for approximately 11 to 17 days (Lechien et al. 2020), there is abundant evidence that such symptoms can become chronic in some patients. COVID-19 survivors commonly report lingering symptoms even after the resolution of the initial infection, now referred to as post-acute sequelae of COVID-19 (PASC) or Long COVID (Ramakrishnan et al. 2021). Recent estimates reveal that approximately 13% of patients with COVID-19 experience persistent symptoms one-month post-infection, and 2.5% post 3 months, with rates greater than 30% among hospitalized patients with severe acute disease (Iqbal et al. 2023; Ballouz et al. 2023). Although other viral illnesses may also present with post-viral sequelae. Global analysis revealed that the cumulative prevalence of sequelae of COVID-19 is 6 times higher than other viral illnesses (mononucleosis, and pneumonia) making Long COVID a public health concern (Lippi et al. 2023; Ho-Yen 1988).

Long COVID is a chronic, multi-system condition that heterogeneously affects multiple parts of the body associated with systemic inflammation, brain fog, depression, joint pain, and loss of appetite. Other common manifestations include fatigue, fever, dyspnea, insomnia, arthralgia, abdominal pain, and diarrhea (Almas et al. 2022; Thaweethai et al. 2023; Choudhury et. 2022). Persistent presence of SARS-CoV-2 and prolonged viral shedding is hypothesized to mediate: (a) direct cell damage; (b) continuous activation of inflammatory cells; (c) induction of autoantibodies against chemokines/cytokines (Muri et al. 2023); and (d) overall activation of the immune system (Umesh et al. 2022). Evidence suggests that Long COVID patients often have abdominal discomfort and dysbiosis (Hilpert and Mikut 2021). Gut dysbiosis can cause changes in blood metabolites such as amino acids, fatty acids, and bile acids, which may contribute to systemic inflammation in Long COVID patients (Gou et al. 2021).

The gut-brain axis is a bidirectional connection between the gut and the brain, which is significantly modulated by the gut microbiota. Such disruption in this axis is observed in Irritable Bowel Syndrome (IBS) (Mukhtar et al. 2019) and Inflammatory Bowel Disease (IBD) (Günther et al. 2021). Moreover, it may also be linked with cognitive and neurodegenerative disorders such as autism spectrum disorder (ASD) (Yu and Zhao 2021), Parkinson’s, (Klann et al. 2021) and Alzheimer disease (Megur et al. 2020). Hence, this gut-brain axis plays a role in the development of multiple disorders across both physiological and neurological domains (Bicknell et al. 2023). It has been hypothesized that dysfunction of the gut-brain axis may be responsible for the cognitive manifestations of Long COVID. This can be explained by the gut microbiome, which, either directly, or through its metabolites such as SCFAs, regulates brain homeostasis. Perturbations of gut homeostasis may also impact signs of mood swings such as pleasure, anxiety, or disgust (Mayer 2011).

Hence, both persistent Gastrointestinal (GI) symptoms and chronic neurological symptoms with concomitant changes in gut microbiome suggest long-term dysbiosis leading to pathologic microbiota-gut-brain (MGB) axis signaling in Long COVID patients (Gareau and Barrett 2023). Therefore, our review aims to explore the possible role of the gut microbiome in the acute and chronic phases of COVID-19. Gut dysbiosis is not limited to GI disturbances but also leads to enhanced production of inflammatory cytokines and lack of nutrients triggering a systemic inflammatory response mainly observed in post-viral sequelae. In this context, the current role of food supplements and probiotics may alleviate the GI symptoms of Long COVID. The understanding of microbiota-based therapeutics for the treatment of Long COVID is under-studied and should be investigated further.

## Case definitions, manifestations, and epidemiology

Long COVID presents a diverse range of multi-organ symptoms and varying severity, posing a challenge to precisely define this syndrome (Szabo et al. 2023). Although a multitude of different definitions and models have been set forward for Long COVID (Table 1), there is a lack of standardized definition among healthcare professionals (Akbarialiabad et al. 2021). A well-known definition by the World Health Organization (WHO) states that Long COVID, or post-COVID-19 condition, is a clinical syndrome characterized by the development or continuation of symptoms, 3 months after the initial infection, which persists for at least 2 months without any alternative explanation (Lippi et al. 2023; Groff et al. 2021; National centre for health statistics 2022; Post COVID-19,2024). This definition was created through a protocol-based Delphi consensus (Soriano et al. 2022), and following this, a set of outcomes were also documented to supplement the definition and assist in diagnosis. The “Core Outcome Set” was defined based on Delphi consensus for adults with post-COVID-19 condition. These include specific symptoms about the functioning of cardiovascular, respiratory, nervous system, cognitive, mental health, and physical health-related outcomes (Munblit et al. 2022). Aside from this, another commonly used guideline was set forth by the National Institute for Health and Care Excellence (NICE), which states that if the duration of symptoms is for 4-12 weeks after acute infection, it is referred to as ongoing symptomatic COVID-19, and if >12 weeks it is referred to as post-COVID-19 syndrome. Both of them are known as Long COVID (NICE 2020).

**Table 1.** Proposed definitions for long COVID

Organization/author	Case definition	Term	References
Center for Disease Control (CDC)	Ongoing symptoms experienced for at least 4 weeks after the initial infection.	Long COVID	National centre for health statistics (2022), Post COVID-19 (2024)
WHO	A clinical syndrome characterized by the development of symptoms 3 months after the initial infection, which persist for at least 2 months without any alternative explanation.	Post-COVID-19 condition	Soriano et al. (2022)
NICE	Symptom duration for 4–12 weeks after acute infection (ongoing symptomatic COVID) or lasting longer than 12 weeks (post COVID-19 syndrome), are both classified as Long COVID.	Ongoing symptomatic COVID, post-COVID-19 syndrome	NICE (2020)
Bala Munipalli et al	PASC: Symptoms persisting beyond the initial 4-week infection period. PACS: Symptoms duration continuing beyond 4 weeks, up until 12 weeks from acute infection.	Post-acute sequelae of COVID-19 (PASC), Post-acute COVID-19 syndrome (PACS)	Munipalli et al. (2022)
Cesar Fernandez-des-las-Penas et al	Transition phase: up to 4–5 weeks Acute post-COVID symptoms: 5–12 weeks Long post-COVID symptoms:12–24 weeks Persistent post-COVID symptoms: greater than 24 weeks	Transition phase, acute post-COVID symptoms, Long post-COVID symptoms, persistent post-COVID symptoms	Fernández-De-las-Peñas et al. (2021)
Researching COVID to Enhance Recovery (RECOVER) cohort	WHO's definition of PASC	Post-acute sequelae of COVID- 19 (PASC)	Thaweethai et al. (2023)

The integrative model proposed a working definition of Long COVID which considered several factors such as individual predisposing factors, biological factors, disease severity factors (hospitalization and treatment), and external factors (psychosocial and COVID-19 associated factors) that promote Long COVID (Fernández-de-las-Peñas et al. 2021). Fernandez-des-las-Penas et al further categorized Long COVID based on extrinsic and intrinsic factors for hospitalized, non-hospitalized and asymptomatic patients, as “Transition” (Acute COVID-19, 4–5 weeks), “Phase 1” (Acute post-COVID, 5–12 weeks), “Phase 2” (Long post-COVID, 12–24 weeks) and “Phase 3” (Persistent post-COVID, >24 weeks) (Table 1) (Fernández-de-las-Peñas et al. 2021a, b).

A consensus definition of the nomenclature is necessary to report, diagnose, treat, and formulate policies regarding and management of Long COVID patients (Soriano et al. 2022). The heterogeneity and severity of long-term symptoms are prone to misleading and incorrect diagnoses. Long COVID may also be overlooked due to unawareness of multiple syndromes encompassing Long COVID (Desgranges et al. 2022; Sigfrid et al. 2021). A standardized definition for Long COVID may aid in better identification and subsequent management of patients earlier in the course of the disease.

The manifestations of Long COVID are diverse and depend on multiple factors, which include geographical location, variant types, pre-existing conditions, and most importantly older age and female gender (Ballouz et al. 2023). Various studies [North America (USA), Europe (Italy), and South Asia (Pakistan, Bangladesh, and India)] identified the most common symptoms of Long COVID as generalized fatigue, dyspnea, myalgia, cough, and headache (Table 2). However, the frequency, severity, and type of Long COVID manifestations exhibit variation across the globe (Table 2). A meta-analysis of 10,945 cases of SARS-CoV-2 infection at 6–12 months follow-up, reported pooled prevalence of muscle weakness (54%), fatigue (30.94%), dyspnea (27.06%), anxiety (25.19%), sleep difficulty (24.11%), difficulty concentrating (22.47%) limited mobility (21.81%,) chest tightness (21.18%), and depression (20.16%). After 12 months, along with the above symptoms, additional manifestations continued including myalgia and joint pain (34.52%), rhinorrhea (30.93%), and neurological symptoms (23.85%) (Ma et al. 2022). A large-scale analysis done on 9764 individuals from the RECOVER cohort reported the presence of 37 symptoms, 6 months post-infection. The most common symptoms were post-exertional malaise (PEM) (87%), fatigue (85%), brain fog (64%), dizziness (62%), GI manifestations (59%), and palpitations (57%) (Thaweethai et al. 2023). Based on previous studies, it can be concluded that certain symptoms are common between South Asian and Caucasian populations such as fatigue, dyspnea, and depression. However, certain manifestations are more prevalent in South Asian populations such as cough and headache (Table 2). The regional variation of symptoms within the South Asia population and underlying pathophysiological mechanisms warrants further investigation to formulate prevention and treatment strategies for Long COVID.

**Table 2.** Most common manifestations of long COVID across different regions

Symptoms	Italy ^Carfi et al. (2020)^ (n=143)	US RECOVER ^Thaweethai et al. (2023)^ (n=8646)	Pakistan ^Qamar et al. (2022)^ (n=331)	India ^Anjana et al. (2021)^ (n=154)	Bangladesh ^Mahmud et al. (2021)^ (n=355)
	2 months	>6 months	1 month	3 months	–
Fatigue	53.1%	38%	–	5.8%	33%
Smell abnormalities	< 20%	13% (taste and smell combined)	17.2%	–	2%
Taste abnormalities	9%	–	14.5%	–	–
Brain fog/cognitive dysfunction	–	20%	–	–	0.6%
Headache	< 20%	13%	22.7%	5.8%	3.4%
Shortness of breath	43.4%	11%	9.1%	2.5%	7%
Myalgia	< 20%	14%	39.9%	3.2%	0.6%
Cough	< 20%	12%	30.2%	0.6%	8.5%
Joint pain	27.3%	17%		2.5%	1.4%
Chest pain	21.7%	8%	12.7%		0.8%
Mood disturbances	–	–	32.6%	–	–
Loss of appetite	< 20%	–	13%	–	–
Diarrhea	< 20%	25%	8.2%	–	–
Sore throat	< 20%		–	–	–
Fever	–		8.8%	–	–

### Gut-brain axis in Long COVID disease

Our current understanding of functional GI disorders encompasses several overlapping symptoms (Drossman and Hasler 2016), such as dysfunction in intestinal barrier function is linked with changes in gut microbiota, and exacerbation of abdominal pain due to stress in IBS as a result of abnormal response to the Hypothalamic-Pituitary-Adrenal (HPA) axis. The gut-brain axis may have a central importance in Long COVID, not only because of communication between two organs, but neurological manifestations observed in Long COVID may be linked to gut dysbiosis (Bicknell et al. 2023; Arneth 2018). Along with common Long COVID symptoms such as fatigue (37%), brain fog (32%), and memory impairment (28%), frequently reported neuropsychiatric manifestations, were sleep disturbances (31%), anxiety (23%), and depression (17%) (Premraj et al. 2022). In a systematic review, 22% of study participants manifested GI-related symptoms during Long COVID, compared to 12% in the acute phase of the disease. Among those, 10% had loss of taste, 9% had loss of appetite, 7% had abdominal pain, 6% had nausea/vomiting, and 5% had diarrhea (Choudhury et al. 2022). Therefore, both GI and neurological symptoms are prevalent among Long COVID patients, and among other mechanisms it is hypothesized to be through dysregulation of the gut-brain axis, probably driven by dysbiosis of the gut microbiota (Ancona et al. 2023).

## Risk factors of long COVID

Multiple studies have reported clinical and epidemiological risk factors associated with Long COVID. Among those, age (Logue et al. 2021), female sex (Takahashi et al. 2020), obesity (Szabo et al. 2023), type 2 diabetes mellitus (Raveendran and Misra 2021), severe acute SARS-CoV-2 infection (Nalbandian et al. 2021), and nutrient deficiency (Raveendran and Misra 2021) were major risk factors of Long COVID post-acute infection. Furthermore, minor risk factors include the presence of autoantibodies (Muri et al. 2023; Su et al. 2022a), Epstein-Barr virus (EBV) reactivation (Gold et al. 2021), and the healthcare profession (Whitaker et al. 2022). In addition, ethnicity has been reported to be one of the epidemiological risk factors associated with the development of Long COVID (Nalbandian et al. 2021).

### Age

Generally Long COVID symptoms manifest with older age, and a larger proportion of older people suffer from this disease. More than 40% of people over 65 years experienced prolonged symptoms, compared with 26–30% between the ages of 18 to 64 years (Logue et al. 2021). Older patients commonly report symptoms like cough, arthralgia, fatigue, and dyspnea. This may be linked to “inflamm-aging”, a pro-inflammatory state associated with aging (Daitch et al. 2022). However, an association of Long COVID with inflammation is not limited to older age, as adolescents with pre-existing multisystem inflammatory syndrome (MIS), also experience Long COVID (Ciarambino et al. 2021; Zheng et al. 2023). A meta-analysis showed a prevalence (25.24%) of symptoms like dyspnea, fatigue, and headache in both children and adolescents (Zheng et al. 2023; Perumal et al. 2023). The significant association of Long COVID with older age groups could also be due to the severity of infection and co-morbidities.

### Gender

A strong association was reported between older age and female gender, which may relate to decreased estrogen levels in post-menopausal women. A cohort study on 418 patients reported an increased risk of obese women (57%) with persistent symptoms of Long COVID (Desgranges et al. 2022). Distinct gender-related patterns were observed in outcomes of Long COVID, and these differences may be attributed to underlying gender differences in immune phenotypes (Takahashi et al. 2020). Among males with more severe acute infection, there were higher levels of pro-inflammatory cytokines including IL-8, IL-18, and CCL5, and lower levels of CD8+ T cells. However, females with more severe diseases had higher levels of innate immune cytokines including IL-15, M-CSF, and CXCL10. Older females had a more robust T cell activation response initially, and more intact T cell function compared to age-matched males. Females had a higher level of IFNγ responses from CD8+ T cells, compared to males, who showed a declining trend with age. Moreover, sex-related disparities in immune responses and outcomes in SARS-CoV-2 infection may have broader implications for understanding gender-specific health conditions (Takahashi et al. 2020; Perumal et al. 2023).

### Metabolic syndrome

#### Type 2 diabetes mellitus

Pre-existing type 2 diabetes mellitus increases the risk of Long COVID. Poorly controlled diabetes has been associated with increased severity of acute COVID-19 infection, subsequent hospitalization, and prolonged recovery from disease (Raveendran and Misra 2021; Huang et al. 2020). This may be due to the underlying inflammatory state present in diabetic patients, along with the initial suppression of CD8+ T cells (Bergamaschi et al. 2021), which aggravates SARS-CoV-2 infection, leading to prolonged recovery (Raveendran and Misra 2021).

#### Obesity/overweight

Obesity is another risk factor for developing Long COVID. A retrospective cohort study in health care workers reported a higher risk in individuals with BMI >25.9 kg/m^2^ (Vimercati et al. 2021), indicating that obesity may be a predisposing factor for Long COVID (Scherer et al. 2022). Increased viral persistence, adiposity due to chronic inflammation, insulin resistance, and higher levels of ACE-2 expression are possible mechanisms of Long COVID predisposition (Desgranges et al. 2022).

#### Ethnicity

Among other epidemiological factors, ethnicity also plays a significant role in influencing the incidence, severity, presentation, and outcomes of Long COVID (Perumal et al. 2023). A cohort study of 486,149 non-hospitalized, confirmed COVID-19 cases revealed that ethnicity is associated with the risk of development of Long COVID symptoms. Blacks, mixed ethnicities (including participants from Bangladesh, India, Pakistan, and East Asia), and ethnic minority groups (Middle Eastern, Native American) were found to be more susceptible to Long COVID as compared to white population (Subramanian et al. 2022). Moreover, a study conducted in the US showed that Black participants experienced higher rates of dyspnea, myalgia, and arthralgia compared to other ethnicities (Yomogida et al. 2021).Racial/Ethnic minority groups experienced more Long COVID symptoms and faced additional barriers in seeking medical support that hindered medical referral and counseling, mainly due to a lack of trust in health professionals, language barriers, poor understanding of the disease, and certain religious and cultural beliefs (Smyth et al. 2022). Therefore, the connection between ethnicity, incidence, and severity of Long COVID is not fully understood (Lavery et al. 2020).

#### Severity of acute infection

To understand how the risk factors of severe acute infection of SARS-CoV-2 affect Long COVID development, 327 acute COVID-19 patients of differing severity were followed in one study, who received mechanical ventilation during acute infection. Patients who were admitted to the Intensive Care Unit (ICU) or received mechanical ventilation had 3.6 times higher risk of persistent symptoms and higher dyspnea during follow-up (Sigfrid et al. 2021). Mechanical ventilation and ICU admissions were positively associated with the development of Long COVID (Nalbandian et al. 2021). Hospitalized COVID-19 patients manifested post-intensive care syndrome (PICS), which exacerbates Long COVID symptoms, leading to longer recovery in the post-acute phase (Vrettou et al. 2022). Severe acute infection is a well-established risk factor for Long COVID, however, individuals who had prior asymptomatic or mild COVID-19 infection may also suffer from Long COVID. A cohort study on laboratory-confirmed, non-hospitalized COVID-19 patients also showed the persistence of one or more symptoms up to 30 days after the initial diagnosis (Bell et al. 2021).

## Normal composition of the gut microbiota

Our GI tract is home to commensal bacteria, fungi, archaea, and viruses. These organisms are collectively known as the ‘gut microbiota’ (Thursby and Juge 2017). The group of microorganisms that attracts the most attention is gut bacteria, because of their significant diversity, richness, and accessibility. Gut bacteria tend to increase in diversity and density moving along the gut [(stomach (10^1^ bacteria per gram), duodenum (10^3^/g), jejunum (10^4^/g), ileum (10^7^/g), and colon (10^12^/g)] (Dieterich et al. 2018). The most abundant phyla include *Bacteroidetes*, *Firmicutes,* *Actinobacteria*, and *Proteobacteria,* with lower levels of *Verrumicrobia*, *Acidobacteria*, and *Fusobacteria* (Dieterich et al. 2018; Rajilić-Stojanović and Vos 2014). The fecal microbiota has more bacterial diversity compared to upper GI microbiome (duodenal and mucosal). Lactobacilli, Veillonella predominate in the proximal gut, Bacilli (Firmicutes), Streptococcaceae (Firmicutes), Actinomycinaeae, and Corynebacteriaceae in the duodenum (Frank et al. 2007), while the genus *Bifidobacterium,* predominates in the colon (Sekirov et al. 2010; Vaga et al. 2020). Microbiota in early life follow a post-natal program of microbiota assembly (Subramanian et al. 2014), which constantly changes in the first two years of life (Stewart et al. 2018). This postnatal program of assembly is highly influenced by exposure to antibiotics (Vangay et al. 2015), weaning diets (Koenig et al. 2011), and other factors such as delivery mode (Dominguez-Bello et al. 2010) and contact with siblings and pets (Ronan et al. 2021). The gut microbiome is dynamic and constantly modified in interaction with the external environment.

### Role of SARS-CoV-2 infection in disrupting normal ACE2 and gut microbiota function in long COVID

**Fig. 1:**
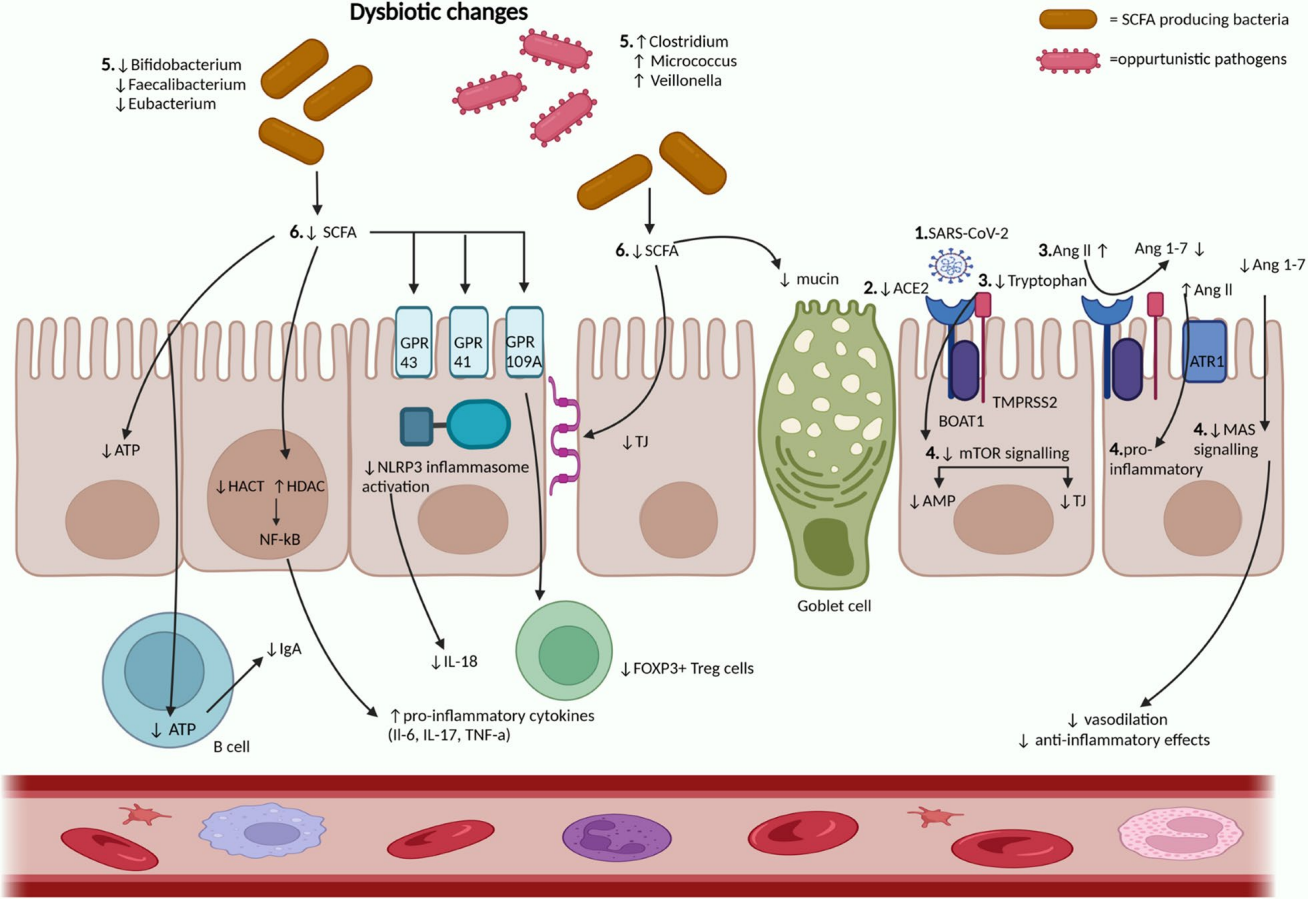
The suggested mechanism of possible gut dysbiosis and persistent SARS-CoV-2 infection in Long COVID. Long COVID-mediated ACE2 dysfunction is characterized by the persistent presence of SARS-CoV-2 (1), which leads to internalization of the ACE2-B^0^AT1 complex (2), resulting in decreased tryptophan absorption (3). This leads to decreased mTOR activation which is needed for AMP and TJ formation. Reduced levels of AMPs affect the normal bacterial composition and decreased TJ functioning allows for bacterial translocation, resulting in bacterial imbalance (4). The subsequent dysbiosis is characterized by an increase in the number of opportunistic pathogens, including species belonging to *Clostridium, Micrococcus*, and *Veillonella*, with a decrease in beneficial SCFAs producing bacteria including those belonging to species *Bifidobacterium, Faecalibacterium,* and *Eubacterium* (5). SCFA, namely butyrate, acetate, and propionate, which are reduced in Long COVID, are responsible for maintaining intestinal homeostasis and immune function (6). SCFAs inhibit histone deacetylase (HDAC) while simultaneously activating histone acetylase (HACT). Imbalance in this acetylation process leads to dysregulation of NF-Kb function, resulting in increased production of pro-inflammatory cytokines. SCFA act on their G-protein coupled receptors (GPR43, GPR41, GPR109A) to carry out anti-inflammatory actions. Decreased activation of these pathways include reduced NLRP3 inflammasome activation, and hence reduced IL-18, needed for innate immunity. Levels of FOXP3 regulatory T cells are also decreased. SCFA typically act as energy sources for intestinal epithelial cells and B cells. Reduced levels lead to less energy for the synthesis of mucosal IgA antibodies by the B cells. Intestinal permeability is increased due to reduced TJ formation, and mucin formation by the goblet cells is also reduced because of decreased SCFA. Additionally, these effects due to decreased SCFA are exacerbated by reduced ACE2 (2), which leads to reduced conversion of Ang ll to Ang 1-7 (3), resulting in pro-inflammatory effects and tissue injury (4). SCFA (Short chain fatty acid), HDAC (histone deactylase), HCAT (histone acetylase), TJ (tight junctions), AMP (antimicrobial peptide), TMPRSS2 (Trans membranous protease serine 2), NLRP3 (NOD-, LRR- and pyrin domain-containing protein 3)

The composition of the gut microbiome is highly influenced by diet, lifestyle, and metabolic diseases (Lee et al. 2022). SARS-CoV-2 infection is known to disrupt normal gut microbiota and gut homeostasis (Fig. 1).

There is substantial evidence for the presence of SARS-CoV-2 Nucleocapsid protein in gastric biopsies (Hany et al. 2024), and abundant ACE2 receptor distribution in the small intestine (Qi et al. 2020; Ning et al. 2022). In a study of 14 asymptomatic individuals, upper and lower GI biopsies were taken approximately 4 months (range of 2.8 to 5.7 months) after the initial SARS-CoV-2 infection. Of those 14, 5 had N protein detected on biopsy tissues, while 3 had amplicon sequences verified as SARS-CoV-2 virus persistence at an extra-pulmonary site (Gaebler et al. 2021). Similarly, a longitudinal analysis done on 673 stool samples from 113 COVID-19 patients, over 10 months, revealed 12.7% shedding of SARS-CoV-2 RNA in the feces at 4 months after the first positive test, and 3.8% up to 7 months. The presence of fecal RNA correlated with the presence of GI symptoms, indicating an ongoing infection (Natarajan et al. 2022).

The presence of persistent SARS-CoV-2 virus or viral antigens is considered as a risk factor for developing Long COVID. A group of researchers at Yale University described the multitude of hypotheses about Long COVID disease, including viral persistence, activation of latent viral infection (EBV), gut dysbiosis, and cross-reactive autoantibodies (Vojdani et al. 2023; Klein et al. 2022). Gut dysbiosis has multiple downstream effects including activation of inflammatory cells, increased cytokine levels, and damage to the GI barrier. Gut dysbiosis is a major cause of inflammation, which may be causally related to persistent symptoms observed in Long COVID. Similarly, underlying inflammation observed in obese individuals is thought to be linked with gut dysbiosis. A cohort study done on Long COVID patients showed that obese individuals had lower alpha diversity and higher pro-inflammatory markers (FGF2, IL-6, IFNγ, MCP-1) with attenuation of viral neutralization capacity through activation of High Mobility Group Box 1 (HMGB1). The study concluded that obese individuals had disturbed microbiome-immune axis, which may contribute to culminates in Long COVID disease (Rubas et al. 2024).

ACE and ACE2 have been implicated in many human pathologies such as cardiovascular disease, lung injury, and renal diseases. In the context of SARS-CoV-2, ACE2 acts as a receptor for binding, and it is essential for neutral amino acid transporters in the gut. Disruption of ACE2 is manifested with diarrhea and dysbiosis of the gut (Perlot and Penninger 2013). It is proposed that the virus gains entry into enterocytes via ACE2, with the facilitation of host receptor trans membranous protease serine 2 (TMPRSS2) (Devaux et al. 2021). SARS-CoV-2 causes the internalization of the ACE2/B^0^At1 complex (Devaux et al. 2021), thereby reducing its expression on the intestinal epithelial cells, as seen in other chronic inflammatory conditions such as Inflammatory bowel disease (IBD) (Burgueno et al. 2020), motility disorder (Coles et al. 2022) and microbial dysbiosis due to higher levels of ACE2 and TMPRSS2 in the GI tract (Wang et al. 2022). ACE2 is a chaperone for the sodium-dependent amino acid transporter B^0^AT1, which is primarily responsible for the absorption of tryptophan and glutamine (Wang et al. 2022). These amino acids are necessary to strengthen the intestinal barrier and decrease pro-inflammatory cytokines (Perlot and Penninger 2013). The downregulation of B^0^AT1 by SARS-CoV-2 in intestinal epithelial cells is suggestive to be one of the mechanisms of microbial dysbiosis (Wang et al. 2022). Absorption of tryptophan leads to mammalian target of rapamycin (mTOR) activation, which enhances antimicrobial peptide synthesis by Paneth cells to maintain gut homeostasis and limits enteropathogens’ colonization (Oliveira et al. 2020). Additional effects of mTOR signaling include increased expression of tight junction proteins such as Occludin, Claudin-4, and Zonulins (ZO-1, and ZO-2) in the intestinal cells, which contribute to detrimental pathogen entry (Wang et al. 2015; Carvalho et al. 2021).

## Changes in the gut microbiota composition

### Dysbiosis during acute COVID-19

During acute COVID-19 infection, levels of presumed beneficial bacteria belonging to *Ruminococcus, Faecalibacterium Roseburia, Lachnospira, and Prevotella* were found to be reduced, while the levels of presumed opportunistic pathogens belonging to *Streptococcus*, *Enterococcus*, and *Corynebacterium* were elevated (Cheng et al. 2022). Contrary to opportunistic pathogens, Faecalibacterium produces anti-inflammatory molecules such as salicylic acid (Sokol et al. 2008) beneficial for gut health (Tian et al. 2021). Additional studies suggest a higher abundance of *Clostridium hathewayi*, *Actinomyces viscosus,* and *Bacteroides nordii* in COVID-19 (Table 3). *Alistipes onderdonkii* and *Faecalibacterium prausnitzii* were found to be anti-inflammatory, whereas *Clostridium hathewayi* correlated with disease severity (Hilpert and Mikut 2021*).*

**Table 3.** Alterations in the gut microbiota vs. time during recovery from COVID-19

Microbiota association with COVID-19 recovery	COVID-19 Severity	Correlation Virus Fecal Shedding	Relative Abundance COVID-19	6 Month PASC Association	1 Year Post COVID-19
*Akkermansia muciniphila *Yeoh et al. (2021)					
*Bacteroides vulgatus* Liu et al. (2022a); Yeoh et al. (2021)					
*Bacteroides dorei* Yeoh et al. (2021), Zuo et al. (2020)					
*Bacteroides thetaiotaomicron *Chen et al. (2022)					
*Bacteroides massiliensis *Chen et al. (2022)					
*Bacteroides ovatus *Chen et al. (2022), Yeoh et al. (2021)					
*Bacteroides caccae *Yeoh et al. (2021)					
*Copraobacillus *Chen et al. (2022)					
*Clostridium ramosum *Chen et al. (2022)					
*Clostridium hathewayi *Chen et al. (2022)					
*Ruminococcus gnavus *Liu et al. (2022a; Yeoh et al. (2021)					
*Ruminococcus torques* Yeoh et al. (2021)					
*Erysipelatoclostridium *Liu et al. (2022a)					
*Ramosum Clostridium bolteae *Liu et al. (2022a)					
*Clostridium innocuum *Liu et al. (2022a)					
*Eubacterium hallii* Zhan et al. (2023)					
*Subdoligranulum *Zhan et al. (2023)					
*Ruminococcus *Zhan et al. (2023)					
*Dorea *Zhan et al. (2023)					
*Coprococcus *Zhan et al. (2023)					
*Eubacterium ventriosum *Zhan et al. (2023)					
*Fusicatenibacter *Cui et al. (2022)					
*Agathobacter Lachnospiraceae* Cui et al. (2022)					
*Faecalibacterium *Cui et al. (2022)					

Liu et al. (2022), Yeoh et al. (2021), Zuo et al. (2020), Cui et al. (2022), Zhan et al. (2023), Chen et al. (2022)

Positive correlation/increased levels

Inverse correlation/decreased levels

No information

### Dysbiosis during post-COVID-19 (3–6 months)

A study performed on 133 patients at 3 and 6 months post-acute COVID infection revealed that those suffering from Long COVID had a less diverse gut microbiome, with lower abundance of SCFAs producing bacteria including *Bifidobacterium adolescentis, Faecalibacterium prausnitzii,* and *Lachnospiraceae.* Long COVID individuals had a higher abundance of Candida albicans and Pseudomonas phages Pf1, with predominance of Klebsiella spp. (Liu et al. 2022a; Simadibrata et al. 2023). Post-COVID patients showed lower levels of SCFAs and L-isoleucine, as a consequence of the loss of presumed beneficial bacteria involved in many biochemical pathways (Zhang et al. 2022a). The persistent impact of SARS-CoV-2 infection on the gut microbiome was observed up to 6 months, with a higher abundance of opportunistic pathogens such as *Erysipelatoclostridium ramosum*, *Clostridium bolteae*, and *Clostridium innocuum*. The occurrence of such bacterial taxa was associated with a higher viral load and increased severity markers. Interestingly such changes in post-acute COVID-19 syndrome (PACS) were relatively stable over time (3 and 6 months) (Liu et al. 2022a). The gut microbial diversity was not fully restored until six months or longer post-acute illness. Low microbial diversity, measured through the Chao1 index, in the post-acute convalescence phase was also correlated with high inflammation scores (Chen et al. 2022).

### Dysbiosis during post-COVID-19 (1-year)

Dysbiosis in microbial composition was pronounced in the cohort of 84 recovered patients from COVID-19, one-year post-illness. A significant decline was observed in SCFA-producing bacteria, notably *Eubacterium.hallii_*group, *Subdoligranulum*, *Ruminococcus*, *Dorea*, *Coprococcus*, and *Eubacterium.ventriosum_*group. These bacterial taxa also showed a positive correlation with lymphocytes but a negative correlation with CRP (Ruminococcus), neutrophil count (Dorea), and natural killer cells (NK cells) (*Eubacterium.ventriosum_*group, Ruminococcus) (Table 3) (Zhan et al. 2023).

## Changes in the gut microbiome composition and relationship with long COVID symptoms

Aside from general changes in the gut microbiome observed in Long COVID, certain alterations in gut microbiome composition correlate with the main persistent symptoms of Long COVID such as fatigue, poor memory, and hair loss (Liu et al. 2022b). Respiratory symptoms were manifested with increased abundance of *Streptococcus anginosus, Streptococcus vestibularis,* and *Streptococcus gordonii *(Liu et al. 2022b). Increased levels of *Clostridium innocuum* and *Actinomyces naeslundii* were seen in individuals with neurological symptoms (Liu et al. 2022b). Patients with mental stress and depression may have alterations in *Lactobacilli* and *Clostridia* species with the former being significantly reduced (Gao et al. 2022). Patients experiencing hair loss had reduced levels of butyrate-producing bacteria such as *Bifidobacterium pseudocatenulatum* and* Faecalibacterium prausnitzii*. These alterations in the gut microbiome may be explained by underlying changes in the gut barrier and immune dysfunction, and possibly linked with microbial dysbiosis (Fig. 1) (Zhang et al. 2023).

### Role of microbiota-gut-brain (MGB) axis in long COVID

The gut-brain axis has a complex bidirectional relationship involving neural, hormonal, immune, and metabolic pathways that regulate physiological processes such as emotion and gut motility (Mayer 2011). Gut microbiota, through microbial metabolites, modulate signaling processes in the brain via the MGB axis (Bicknell et al. 2023).

**Fig. 2:**
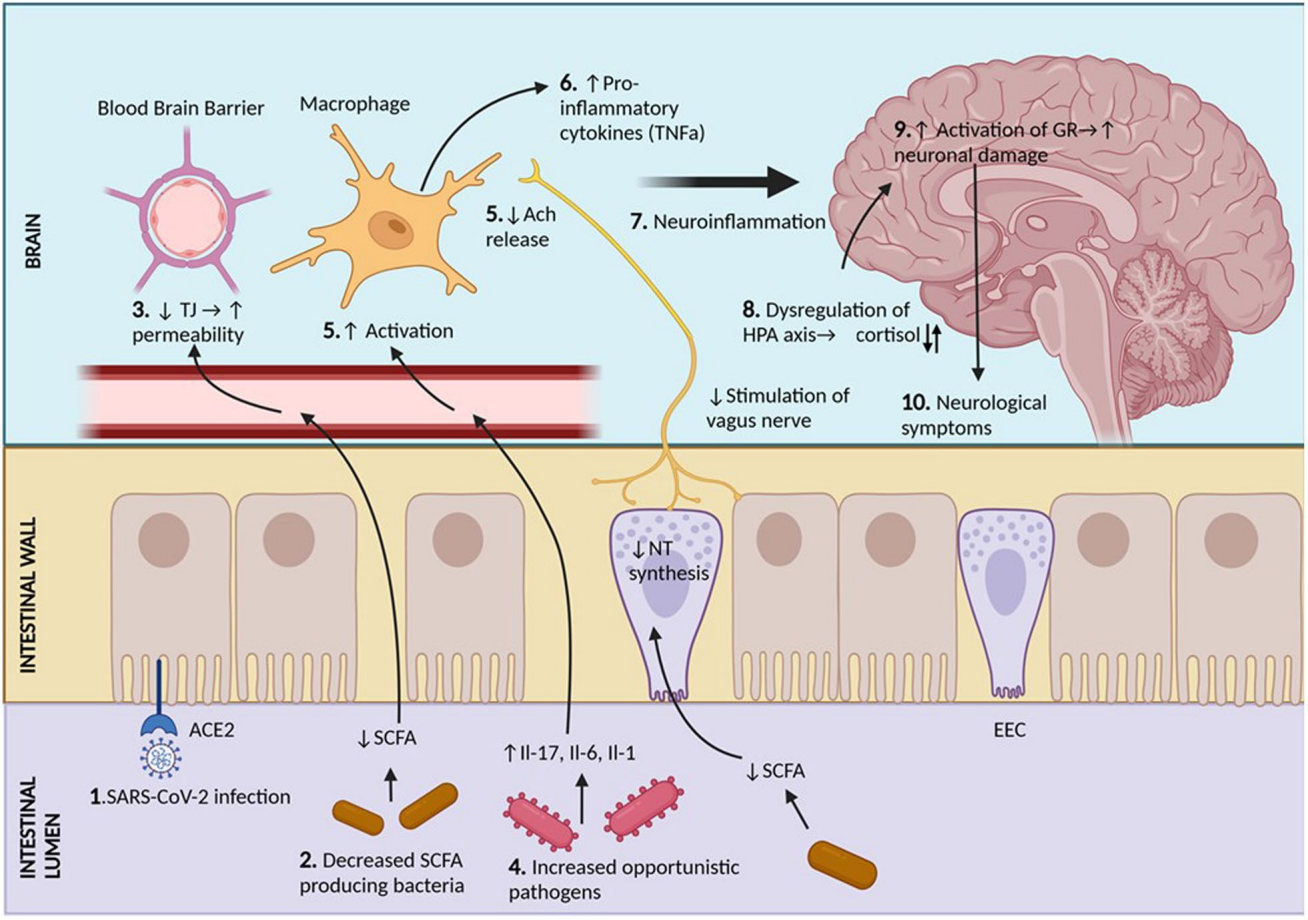
Contribution of microbiota-gut-brain-axis in causing the neurological symptoms of Long COVID. Persistent levels of SARS-CoV-2 in the gastrointestinal wall (1) leads to reduced production of SCFA, as discussed earlier (2). Decreased serum levels of SCFA results in lower production of TJ for the blood-brain barrier, resulting in increased permeability of the brain capillaries (3). As a result, pro inflammatory cytokines [IL-17, IL-6, and IL-1] produced in the gut by opportunistic pathogens (4) can cross the blood brain barrier and activate resident macrophages (5). At the same time, reduced SCFA results in reduced neurotransmitter production in the enteroendocrine cells. This leads to reduced stimulation of the vagus nerve afferents, and consequently, less Ach released on the macrophages. Ach is responsible for inhibiting the production of TNF-α by macrophages. Thus, the collective effect of the opportunistic pathogens and reduced vagal stimulation leads to increased proinflammatory cytokine production by the macrophages (6). The resultant neuroinflammation (7) of the brain parenchyma affects the hypothalamus, which affects the HPA axis (8). Cortisol production increases, which acts in the prefrontal cortex and hippocampus due to their abundant GR (9). This affects synaptic connection and neuronal integrity, which is hypothesized to explain the neurological symptoms of Long COVID, such as depression (10). SCFA (Short chain fatty acids), TJ (tight junctions), NT (neurotransmitter), HPA (hypothalamic pituitary adrenal) axis, EEC (enteroendocrine cell), GR (glucocorticoid receptor), Ach (Acetyl Choline)

Gut dysbiosis is thought to affect the brain functioning through the MGB axis, as often observed in functional gastric disorders such as IBS, gastroparesis, gastroesophageal reflux (GERD) (Mukhtar et al. 2019), and IBD (Günther et al. 2021). It is hypothesized that similar mechanisms may be responsible for the neuropsychiatric and neurological symptoms observed in Long COVID patients (Ancona et al. 2023). The gut-brain axis is illustrated in Fig 2 with potential mechanisms involving gut microbiota-induced neurological symptoms.

The gut dysbiosis-mediated host immune responses to COVID-19 play a significant role in the disruption of the MGB axis. Inflammation in the gut and brain is responsible for neuroinflammation through the MGB axis (Gareau and Barrett 2023; Bostick et al. 2022). Another possible reason could be due to alterations in the HPA axis. The HPA may be influenced by gut dysbiosis and may contribute to the pathogenesis of depression in Long COVID patients.

Long COVID-induced gut dysbiosis is characterized by decreased ACE2 function, leakiness of the gut (Umesh et al. 2022), and reduced levels of SCFA-producing bacteria (Zhang et al. 2022a; Zhan et al. 2023). In the gut, SCFAs are also responsible for maintaining the integrity of the tight junctions of the brain (Tran and Hasan 2021). Studies on germ-free mice reported that perturbation in normal gut microbiota composition leads to reduced expression of Occludin and Claudin-5 proteins, thereby increasing the permeability of the blood-brain barrier. The administration of SCFA-producing bacteria such as *B. thetaiotaomicron* increased the integrity of the blood-brain barrier (Braniste et al. (2014)*.* Therefore, reduced serum SCFAs, as seen in Long COVID (Zhang et al. 2022a), may increase the permeability of the blood-brain barrier. The increased permeability of the intestinal wall and blood-brain barrier may allow pro-inflammatory cytokines, LPS, and toxins to leak out of the gut into the central nervous system and cause neuroinflammation by stimulating microglial cells and through the recruitment of macrophages (Parker et al. 2020). The sources of pathogenic signals in Long COVID may include LPS-producing bacteria (Bicknell et al. 2023), opportunistic pathogens, cytokines (IL-17, IL-6, and IL-1) (Fanelli et al. 2022), and a reduction in the immunomodulatory potential of gut bacteria (Yeoh et al. 2021). This inflammation results in a positive feedback loop that may play a role in the formation of neurofibrillary tangles and amyloid-beta deposition as seen in Alzheimer’s disease (Webers et al. 2020). Neurological symptoms in Long COVID patients may increase the risk for the development of neurodegenerative disease (Bicknell et al. 2023). Three pathways have been described for the MGB axis; neuroinflammation pathway, neuroendocrine pathway, and direct pathway via vagus nerve (Vakili et al. 2022). The vagus nerve plays a role in combatting excessive cytokine production. Mice with deficient α7 Nicotinic Acetylcholine Receptor significantly impaired regulation of TNFα in non-bone-marrow derived cells. For inflammatory reflux, expression of α7 Nicotinic Acetylcholine Receptor in other cells is required, and this pathway is not solely dependent on T cells (Olofsson et al. 2012). These anti-inflammatory actions of the vagus nerve are affected by increased LPS and reduced SCFAs, through Enteroendocrine cells (EECs) that influence the vagus nerve stimulation (Vakili et al. 2022). Some of these neurotransmitters released from EECs may include norepinephrine, dopamine, and serotonin (Strandwitz 2018).

Neuroinflammation is thought to disrupt the HPA axis, which regulates the production of serotonin (Sălcudean et al. 2023).The dysregulation of HPA decreases cortisol levels in Long COVID patients which is not associated with a compensatory increase in Adrenocorticotropic hormone (ACTH) levels(Gaebler et al. 2021). Overall, it is suggested that a dysregulation of cortisol, often seen as hypercortisolemia during the early phase of the disease, may be a risk factor for Long COVID (Su et al. 2022b). As shown in Fig. 2, chronic HPA axis activation resulting from neuroinflammation, cytokines, and microbial antigens leads to excessive glucocorticoid signaling influencing dendritic and synaptic remodeling, impacting myelination and neurotransmitter release in areas expressing glucocorticoid receptors (prefrontal cortex, amygdala, and hippocampus) (Freimer et al. 2022). Multiple pathways of stress-induced activation of the HPA axis are suggested in the brain, including gut microbial dysbiosis, bacterial products, cytokines, and fewer bioactive SCFAs. Therefore, the administration of probiotics may reduce chronic activation of the HPA axis and modulate glucocorticoid signaling. Treatment with psychobiotics such as Lactobacillus plantarum strain PS128 (PS128) has been found to reduce previously elevated serum corticosterone levels and to stabilize neurotransmitters (dopamine and serotonin), strengthening the role of the gut microbiota in neuropsychiatric disorders (Liu et al. 2016). Hence, we hypothesize the decrease in SCFA-producing bacteria, neuroinflammation and subsequent activation of the HPA axis is a possible mechanism linking psychiatric symptoms seen in Long COVID patients.

However, further studies are needed to evaluate the function of individual brain components, along with alterations in gray and white matter volumes, in Long COVID patients. Similarly, data on levels of neurotransmitter, neuropeptide, and gut hormone levels are limited for Long COVID patients. Experimental studies are needed to evaluate their potential as therapeutic targets and the significance of the MGB axis in Long COVID.

## Changes in markers of inflammation

The dysbiotic changes in Long COVID are associated with changes in inflammatory and immune biomarkers, as the gut microbiota plays a crucial role in regulating inflammation. Long COVID immune profiling has presented the activation of TH17-mediated IL-17 and IL-2 with the concomitant low level of anti-inflammatory cytokines (IL4, IL-10) (Queiroz et al. 2022). Similarly, a meta-analysis done on 23 studies evaluating 18 inflammatory and vascular biomarkers showed that CRP, LDH, D-dimer, and leucocytes remained elevated in Long COVID patients, compared with those that recovered, pointing toward vascular immunopathology in Long COVID (Yong et al. 2023). This observation was consistent in short-term PASC (2 months post-discharge) where fatigue was correlated with high levels of Erythrocyte sedimentation rate (ESR), LDH, CRP, and D-dimer, along with the low level of hemoglobin, ferritin, and albumin (Pasini et al. 2021).

Many cytokine changes in acute COVID-19 infection continued to persist in Long COVID. Notably IL-6, a hallmark cytokine of acute COVID-19 (Queiroz et al. 2022) which also contributes to the cytokine storm, and IL-6 remains elevated during sequelae (Yin et al. 2023).

IL-6 cytokine is debatable as progressive decline in IL-6 has been observed in Long COVID or in recovered COVID-19 patients and is therefore considered a marker of the acute phase of the disease (Queiroz et al. 2022; Yong et al. 2023).

Besides IL-6, other cytokines of the acute inflammatory response including IFN-β, Pentraxin 3 (PTX3), IFN-γ, and IFN-λ2/3 were shown to be elevated during Long COVID, up to 8 months post-infection. A set of inflammatory biomarkers was associated with Long COVID in a predictive model with 78.5–81.6% accuracy. This supports the hypothesis that the acute inflammatory processes is a continuum during Long COVID (Phetsouphanh et al. 2022).

However, there are some exceptions. In severe acute COVID-19 infection, levels of type I Interferons (IFN-α and IFN-β) have been reported to be reduced or impaired. In certain severe cases and critical cases, a high viral load and presence of Damage associated molecular patterns (DAMP) or pathogen-associated molecular patterns (PAMPS) overwhelms type I interferon release (Hadjadj et al. 2020). Contrary to certain cases of acute infection, levels of type I interferon [(IFN-β)] and type III IFN [(IFN-λ1)] remain elevated in Long COVID, indicating high activity of plasmacytoid dendritic cells (pDCs) (Galán et al. 2022).

Aside from systemic inflammatory markers, inflammatory biomarkers specific to gut inflammation such as fecal calprotectin, lipopolysaccharide-binding protein (LBP), and fatty acid-binding protein 2 (FABP2) are elevated in Long COVID patients, particularly those with dysbiosis (Zhang et al. 2023; Prasad et al. 2022; Effenberger et al. 2020).

## Immune system-related changes

Alongside alterations in inflammatory markers, changes in immune cell phenotypes and antibody levels have also been observed in Long COVID. A study done on a Spanish cohort of Long COVID patients reported an increase in the total level of CD8+ lymphocytes as a surrogate of antiviral cytotoxic activity, particularly due to increased effector CD8+ T cells. This could be attributed to latent EBV virus reactivation or the aberrant adaptive immune response to chronic infection(Galán et al. 2022; Altmann et al. 2023).

The number of CD3+ lymphocytes expressing markers Programmed Cell Death Protein 1 (PD-1) and T-cell immunoglobulin and mucin domain-3 (TIM-3) were shown to be elevated in Long COVID patients, possibly due to the constant activation of the immune system in response to the persistence of SARS-CoV-2 antigen (Phetsouphanh et al. 2022; Galán et al. 2022). Studies have also shown variable T cell phenotypes associated with presenting symptoms such as GI symptoms had enrichment of cytotoxic CD8+ phenotypes, 2-3 months after the initial infection (Su et al. 2022a).

Aside from T lymphocytes, levels of CD38^+^HLA-DR^+^ myeloid cells, active CD56+ NK cells (Villapol 2020), and plasmacytoid dendritic cells were also elevated in Long COVID (Phetsouphanh et al. 2022). Plasmacytoid dendritic cells have an important role in antigen presentation to T cells, which may relate to the observed increase in T cell levels (Phetsouphanh et al. 2022). An inconclusive role of Tregs in Long COVID has been observed, (Galán et al. 2022; Patterson et al. 2021). The frequency of T-regs has shown to be variable across the spectrum of Long COVID (Haunhorst et al. 2022).

Levels of B cells were increased in Long COVID patients, particularly memory B cells and plasmablasts responsible for producing IgA and IgG, as compared to acute infection, in which IgM-producing plasmablasts were more prevalent (Shuwa et al. 2021).

Along with changes in immune cells, a dynamic response of SARS-CoV-2 specific antibodies has also been observed in Long COVID. For instance, titers of antibodies directed against spike protein receptor binding domain (RBD) were directly related to the severity of acute infection, reflecting the increased activity of B cells during Long COVID (Su et al. 2022a). Autoantibodies were associated with a myriad of chronic inflammatory conditions, including Long COVID. A recent study revealed that autoantibody titers were inversely related to anti-SARS-CoV-2 antibodies at post-convalescence (Dashraath et al. 2020; Lechien et al. 2020), and also correlated with the downregulation of IFN-α, possibly preventing IFN-α from activating B cells to produce anti-SARS-CoV-2 antibodies (Su et al. 2022a). Like immune cells, antibodies also exhibit variation amongst Long COVID patients, depending on their symptoms. For instance, neurological Long COVID was associated with higher anti-nucleocapsid IgG, whereas GI-predominant Long COVID was associated with autoantibodies. Studies have shown that IgG autoantibodies develop against ACE2, particularly in hospitalized patients, that leads to the suppression of ACE2 expression which may blunt its anti-inflammatory effects (Tsilingiris et al. 2023; Hallmann et al. 2023).

## Targeting the gut microbiome for therapeutics

New interventions that target the gut microbiome are attracting attention for the treatment of Long COVID, including the usage of prebiotics, postbiotics (Łoniewski et al. 2022) and dietary alterations (Hashimoto 2023).

### Diet

Long COVID is a multi-organ, chronic inflammatory condition, where dietary alterations may benefit in ameliorating overall health status (Barrea et al. 2022) and improve dysbiosis (Hashimoto 2023). Such alterations in diet aim to target persistent physical and mental symptoms while counteracting inflammation. The Mediterranean diet is well known for its anti-inflammatory effects. This diet is presumably rich in fiber and complex carbohydrates, omega-3 fatty acids, vitamins, and phytochemicals (Tsigalou et al. 2020) which are presumed to be rich in all of the constituents and are known to have anti-inflammatory and immunomodulatory effects (Barrea et al. 2022). A study conducted on 612 people who consumed the Mediterranean diet demonstrated a remarkable decrease in pro-inflammatory markers such as CRP and IL-17 (Ghosh et al. 2020). Consumption of the Mediterranean diet resulted in the shift of microbiota to a higher abundance of SCFA-producing bacteria, *Faecalibacterium. prausnitzii* and *Eubacterium*, which were observed in Long COVID (Liu et al. 2022a; Zhan et al. 2023). Studies on the Mediterranean diet for prevention and treatment of Long COVID have not been reported, however knowing its anti-inflammatory and microbiome-altering qualities, it might be recommended for Long COVID patients.

A large smartphone-based study done on COVID-19 patients administered a plant-based diet showed a decreased risk of severe COVID-19 infection and joint-related complications (Merino et al. 2021). However, studies need to be done to evaluate the effect of diet on inflammation, immune markers, and the gut microbiota composition in Long COVID (Angelidi et al. 2021).

Micronutrients such as vitamins and minerals are necessary to boost immunity. Particularly, vitamin D has anti-viral effects, through induction of the production of cathelicidin defensins (Wang et al. 2004). Vitamin D also supports the gut microbiota by strengthening gut homeostasis through an increase in the ACE2/Ang1-7/MAS/system, promoting the growth of gut-friendly *Bifida* and *Firmicutes* species. Vitamin D supports gut integrity and reduces hyperinflammatory state (Shenoy 2022). A randomized clinical trial performed on hospitalized patients with acute COVID-19 demonstrated reduced disease severity and ICU admission in the treated group (2%), versus the untreated group (50%), after administration of calcifediol (vitamin D) on the day of admission strengthening the relationship between high dose vitamin D and reduced severity of infection (Entrenas Castillo et al. 2020). A cross-sectional retrospective cohort study was done on Long COVID and non-Long COVID patients 6 months after discharge. Vitamin D levels were likely to be lower in Long COVID patients, and vitamin D was the only independent factor associated with the development of Long COVID. Study subjects with lower vitamin D levels at the time of admission and later were found to be at higher risk of neurocognitive symptoms, dysgeusia, and headache (Filippo et al. 2023).

Overall, it is hypothesized that plant-based, antioxidant-rich diets, Mediterranean diets, and vitamin D supplementation may help alleviate the symptoms of Long COVID and alter the gut microbiome (Merino et al. 2021; Filippo et al. 2023).

### Prebiotics and SCFA

Prebiotics can be defined as non-digestible substances, often found naturally, which selectively activate bacteria in the gut to improve health and host function. Common prebiotics contain various types of oligosaccharides such as fructo-oligosaccharides, galacto-oligosaccharides, trans-galacto oligosaccharides (Davani-Davari et al. 2019), and ariboxylan oligosaccharides (Hu et al. 2021). The gut microbiota can ferment these prebiotics into various metabolites, such as SCFAs (Davani-Davari et al. 2019). A study on the consumption of cranberry oligosaccharides as prebiotics demonstrated induction of SCFAs (acetate, propionic acid, and butyrate) from *Lactobacillus* strains (Hotchkiss et al. 2022). Prebiotics serve as a source of energy for the gut microbiota, whereas bacteria administered to modulate the microbiota may be considered probiotics. The synthesis of SCFAs from prebiotic metabolism had a wide range of anti-inflammatory, immune, and metabolic effects (Davani-Davari et al. 2019).

In Long COVID, the intestinal barrier is disrupted, leading to the formation of ‘leaky gut’ (Villapol 2020). Administering prebiotics results in the formation of SCFAs, butyrate, which restores intestinal permeability by increasing tight junction formation (Snelson et al. 2021). Prebiotic inulin did show higher serum glucagon like peptide 2 (GLP-2), and lower concentration of serum zonulin, indicating improved intestinal barrier function. Zonulin is a protein that regulates intestinal permeability and maintains the integrity of tight junctions, whereas GLP-2 is an enteroendocrine-derived peptide that inhibits epithelial cell apoptosis and enhances mucous production (Russo et al. 2012). Similarly, *Bacteroides thetaiotaomicron* requires oligosaccharides as an energy source for mucus synthesis in the intestinal wall (Zafar and Saier 2021), for downregulating ACE-2, and thus limiting viral entry. In severe acute COVID-19 patients, gut dysbiosis is characterized by the reduction in *Bacteroides thetaiotaomicron*, which persisted throughout hospitalization (Zuo et al. 2020b). Administering oligosaccharide prebiotics during both the acute and convalescent phases may support the maintenance of the mucus layer (Cheong et al. 2023).

Both prebiotics and SCFAs modulate anti-inflammatory effects. A recent meta-analysis on probiotics showed a significant decline in the markers of systemic inflammation (CRP, TNF-α and IL-6) (McLoughlin et al. 2017). These biomarkers are elevated in Long COVID, so prebiotic administration may help in the reduction of inflammatory markers and support the growth of beneficial bacteria (Queiroz et al. 2022; Pasini et al. 2021). Aside from prebiotics, in general, there are formulations and drug delivery challenges in administering SCFAs. Long COVID is characterized by a persistent decrease in SCFAs levels (Zhang et al. 2022a; Zhan et al. 2023). It is hypothesized that administering SCFAs such as butyrate may help compensate for the reduction in SCFA-producing bacteria in Long COVID (Hashimoto 2023).

Limited studies have evaluated the effect of prebiotics on Long COVID. Prebiotics are known to enhance the effects of probiotics (Davani-Davari et al. 2019), and ample evidence supports the role of probiotics in restoring the gut microbiome in Long COVID (Zhang et al. 2022b).

### Probiotics

Probiotics refer to live microorganisms, when administered in the required amounts, confer health benefits to the host. Probiotics are often isolated from commensal gut bacteria and are utilized to improve the gut microbiota composition while regulating the gut ecosystem (Hill et al. 2014). They exert their antiviral role through well-known mechanisms either by increasing NK cell activity, Treg cells, antimicrobial peptide production, or by enhancing antibody production (Hu et al. 2021).

In the context of SARS-CoV-2, probiotics may serve as a barrier to viral entry by blocking the active site of ACE receptors (Olaimat et al. 2020). In computational modeling, *Lactobacillus plantarum* probiotics and its metabolites blocked the receptor binding site for RBD (Anwar et al. 2020). Similar processes of viral entry, or viral fusion inhibition have been described with other viruses, such as influenza A (Baindara et al. 2021). Animal studies using *Lactobacillus plantarum* showed enhancement of vaccine-induced antibody production, by activation of T and B cells, and reduction in pro-inflammatory cytokines such as IL-6 and TNF-α (Xu et al. 2021). Another clinical trial evaluated an administration of the probiotic *Limosilactobacillus reuteri*, DSM 17,938 regularly for 6 months in mRNA vaccinated individuals. A higher serum level of anti-RBD IgA was reported in the intervention (probiotic) arm compared to the placebo arm (Forsgård et al. 2023).

Antibiotic administration during acute COVID-19 infection severely affects gut microbiome, an effect which can be restored through probiotics. However, heterogeneity among patients alters the effect of probiotic treatment (Neris Almeida Viana 2022). Fecal metagenomic profiling showed higher levels of antibiotic resistance genes (tetracycline, vancomycin) in bacteria who received antibiotics, along with elevated levels of pathogenic *Klebsiella* in association with Long COVID-19 (Su et al. 2022). Certain strains of *Klebsiella,* such as *Klebsiella quasipneumoniae,* were found to be elevated in patients with severe COVID infection (Liu et al. 2022a). Therefore, the administration of oral probiotics is thought to deplete antibiotic resistance genes, without rebound increase after stopping probiotic administration (Su et al. 2022). Following a recent meta-analysis, there was a 51% reduction in the severity of symptoms experienced by COVID-19 patients, significant improvement in headache, cough, diarrhea, fatigue and cognitive symptoms with the administration of *Lactobacillus*, *Bifidobacterium* species, *Bacillus coagulans*, *Bacillus subtilis*, *Bacillus clausii* (Neris Almeida Viana 2022; Rathi et al. 2021).

Similarly, another study evaluated the effect of a probiotic/prebiotic preparation (containing *Bifidobacteria strains)* on the gut microbiome in COVID-19 patients 5 weeks after infection. These patients had decreased viral load and reduced levels of IL-6, MCP-1, M-CSF, IL-1RA, and TNF-α. Gut microbiota balance was improved, with reduced opportunistic pathogens and increased levels of bacteria such as *Bifidobacteria,* which is normally depleted in Long COVID (Liu et al. 2022a; Zhang et al. 2022b).

Thus, studies suggest probiotics could be utilized in acute COVID-19 to modulate immune responses, and in Long COVID to restore the microbiome and potentially prevent further complications (Su et al. 2022). However, no standardized guidelines are available for the use of probiotics to prevent or treat Long COVID. Evidence from animal and human studies showed some promising outcomes in IBD (Łoniewski et al. 2022), gut dysbiosis (Wu et al. 2021), and boosting antibody responses (Forsgård et al. 2023). However, limited data are available on the efficacy, safety, and adverse effects of probiotics on Long COVID (Kurian et al. 2021).

## Conclusion

In conclusion, the current review underscores the multifaceted nature of Long COVID and its association with various risk factors. Among risk factors, age, gender, comorbidities like Type 2 diabetes, obesity, and ethnicity all appear to have roles in the development and manifestation of Long COVID symptoms (Nalbandian et al. 2021). Older individuals and post-menopausal females seem to be at the highest risk. Thus, the interplay between immune response and gender remains a subject for more exploration (Takahashi et al. 2020). Further investigations into the underlying mechanisms for Long COVID are essential to addressing this emerging health challenge. For instance, certain risk factors, such as severe infection, result in systemic inflammatory changes (namely, the cytokine storm), which can influence the gut microbiota (Kaushik et al. 2022). Evidence suggests that obesity alters gut bacteria via underlying low-grade inflammation and creates dysbiosis (Breton et al. 2022). Further studies are needed to unfold the relationship between various risk factors, dysbiosis, and its long-term effects on Long COVID. Further research is needed to explore the role of diet, prebiotics, and probiotics in the prevention and treatment of Long COVID.

## Data Availability

Not applicable.

## Abbreviations

SARS-CoV-2: Severe acute respiratory syndrome-coronavirus 2
GI: Gastrointestinal
SCFAs: Short chain fatty acids
PICS: Post-intensive care syndrome
ACE2: Angiotensin converting enzyme 2
TMPRSS2: Trans membranous protease serine 2
MGBA: Microbiota gut brain axis
LPS: Lipopolysaccharide
HPA: Hypothalamic pituitary adrenal
EEC: Enteroendocrine
GLP-2: Glucagon like peptide 2
LBP: Lipopolysaccharide-binding protein
FABP2: Fatty acid-binding protein 2
RBD: Receptor binding domain
pDCs: Plasmacytoid dendritic cells
IBD: Inflammatory bowel disease
PTX3: Pentraxin
FGF2: Fibroblast growth factor 2
IBS: Irritable Bowel Syndrome
GERD: Gastro-esophageal reflex disease
DAMP: Damage associated molecular patterns
PAMS: Pathogen associated molecular patterns
TIM-3: T-cell immunoglobulin and mucin domain-3
PD1: Programmed cell death protein 1
HMGB-1: High mobility group Box 1
mTOR: Mammalian target of rapamycin
AMP: Anti-microbial peptide
PEM: Post-exertional malaise

## References

[CR1] Akbarialiabad H, Taghrir MH, Abdollahi A, Ghahramani N, Kumar M, Paydar S, et al. Long COVID, a comprehensive systematic scoping review. Infection. 2021;49(6):1163–86. 34319569 10.1007/s15010-021-01666-x PMC8317481

[CR2] Almas T, Malik J, Alsubai AK, Jawad Zaidi SM, Iqbal R, Khan K, et al. Post-acute COVID-19 syndrome and its prolonged effects: an updated systematic review. Ann Med Surg. 2022;80:103995. 10.1016/j.amsu.2022.103995 PMC9197790 35721785

[CR3] Altmann DM, Whettlock EM, Liu S, Arachchillage DJ, Boyton RJ. The immunology of long COVID. Nat Rev Immunol. 2023;2023:1–17. 10.1038/s41577-023-00904-7 37433988

[CR4] Ancona G, Alagna L, Alteri C, Palomba E, Tonizzo A, Pastena A, et al. Gut and airway microbiota dysbiosis and their role in COVID-19 and long-COVID. Front Immunol. 2023;14:1080043. 36969243 10.3389/fimmu.2023.1080043 PMC10030519

[CR5] Angelidi AM, Kokkinos A, Katechaki E, Ros E, Mantzoros CS. Mediterranean diet as a nutritional approach for COVID-19. Metabolism. 2021;114:154407. 33080270 10.1016/j.metabol.2020.154407 PMC7833284

[CR6] Anjana NKN, Annie TT, Siba S, Meenu MS, Chintha S, Anish TSN. Manifestations and risk factors of post COVID syndrome among COVID-19 patients presented with minimal symptoms–a study from Kerala, India. J Fam Med Prim Care. 2021;10(11):4023. 10.4103/jfmpc.jfmpc_851_21 PMC8797119 35136762

[CR7] Anwar F, Altayb HN, Al-Abbasi FA, Al-Malki AL, Kamal MA, Kumar V. Antiviral effects of probiotic metabolites on COVID-19. J Biomol Struct Dyn. 2020;39(11):1. 10.1080/07391102.2020.1775123 PMC7298884 32475223

[CR8] Arneth BM. Gut–brain axis biochemical signalling from the gastrointestinal tract to the central nervous system: gut dysbiosis and altered brain function. Postgrad Med J. 2018;94(1114):446–52. 30026389 10.1136/postgradmedj-2017-135424

[CR9] Baindara P, Chakraborty R, Holliday ZM, Mandal SM, Schrum AG. Oral probiotics in coronavirus disease 2019: connecting the gut–lung axis to viral pathogenesis, inflammation, secondary infection and clinical trials. New Microb New Infect. 2021;40:100837. 10.1016/j.nmni.2021.100837 PMC7785423 33425362

[CR10] Ballouz T, Menges D, Anagnostopoulos A, Domenghino A, Aschmann HE, Frei A, et al. Recovery and symptom trajectories up to two years after SARS-CoV-2 infection: population based, longitudinal cohort study. BMJ. 2023;381: e074425. 37257891 10.1136/bmj-2022-074425 PMC10230608

[CR11] Barrea L, Grant WB, Frias-Toral E, Vetrani C, Verde L, de Alteriis G, et al. Dietary recommendations for post-COVID-19 syndrome. Nutrients. 2022;14(6):1305. 35334962 10.3390/nu14061305 PMC8954128

[CR12] Bell ML, Catalfamo CJ, Farland LV, Ernst KC, Jacobs ET, Klimentidis YC, et al. Post-acute sequelae of COVID-19 in a non-hospitalized cohort: results from the Arizona CoVHORT. PLoS ONE. 2021;16(8): e0254347. 34347785 10.1371/journal.pone.0254347 PMC8336814

[CR13] Bergamaschi L, Mescia F, Turner L, Hanson AL, Kotagiri P, Dunmore BJ, et al. Longitudinal analysis reveals that delayed bystander CD8+ T cell activation and early immune pathology distinguish severe COVID-19 from mild disease. Immunity. 2021;54(6):1257. 34051148 10.1016/j.immuni.2021.05.010 PMC8125900

[CR14] Bicknell B, Liebert A, Borody T, Herkes G, McLachlan C, Kiat H. Neurodegenerative and neurodevelopmental diseases and the gut-brain axis: the potential of therapeutic targeting of the microbiome. Int J Mol Sci. 2023;24(11):24. 10.3390/ijms24119577 PMC10253993 37298527

[CR15] Bostick JW, Schonhoff AM, Mazmanian SK. Gut microbiome-mediated regulation of neuroinflammation. Curr Opin Immunol. 2022;76: 102177. 35462279 10.1016/j.coi.2022.102177 PMC9167715

[CR16] Braniste V, Al-Asmakh M, Kowal C, Anuar F, Abbaspour A, Tóth M, et al. The gut microbiota influences blood-brain barrier permeability in mice. Sci Transl Med. 2014;6(263):263158–263158. 10.1126/scitranslmed.3009759 PMC4396848 25411471

[CR17] Breton J, Galmiche M, Déchelotte P. Dysbiotic gut bacteria in obesity: an overview of the metabolic mechanisms and therapeutic perspectives of next-generation probiotics. Microorganisms. 2022;10(2):452. 35208906 10.3390/microorganisms10020452 PMC8877435

[CR18] Burgueno JF, Reich A, Hazime H, Quintero MA, Fernandez I, Fritsch J, et al. Expression of SARS-CoV-2 entry molecules ACE2 and TMPRSS2 in the gut of patients with IBD. Inflamm Bowel Dis. 2020;26(6):797. 32333601 10.1093/ibd/izaa085 PMC7188157

[CR19] Carfì A, Bernabei R, Landi F. Persistent symptoms in patients after acute COVID-19. JAMA. 2020;324(6):603–5. 32644129 10.1001/jama.2020.12603 PMC7349096

[CR20] Carvalho T, Krammer F, Iwasaki A. The first 12 months of COVID-19: a timeline of immunological insights. Nat Rev Immunol. 2021;21(4):245–56. 33723416 10.1038/s41577-021-00522-1 PMC7958099

[CR21] Chen Y, Gu S, Chen Y, Lu H, Shi D, Guo J, et al. Six-month follow-up of gut microbiota richness in patients with COVID-19. Gut. 2022;71(1):222–5. 33833065 10.1136/gutjnl-2021-324090 PMC8666823

[CR22] Cheng X, Zhang Y, Li Y, Wu Q, Wu J, Park SK, et al. Meta-analysis of 16S rRNA microbial data identified alterations of the gut microbiota in COVID-19 patients during the acute and recovery phases. BMC Microbiol. 2022;22(1):1–13. 36376804 10.1186/s12866-022-02686-9 PMC9662111

[CR23] Cheong K-L, Chen S, Teng B, Veeraperumal S, Zhong S, Tan K. Oligosaccharides as potential regulators of gut microbiota and intestinal health in post-COVID-19 management. Pharmaceuticals. 2023;16(6):860. 37375807 10.3390/ph16060860 PMC10301634

[CR24] Choudhury A, Tariq R, Jena A, Vesely EK, Singh S, Khanna S, et al. Gastrointestinal manifestations of long COVID: a systematic review and meta-analysis. Therap Adv Gastroenterol. 2022;15:17562848221118404. 36004306 10.1177/17562848221118403 PMC9393939

[CR25] Ciarambino T, Para O, Giordano M. Immune system and COVID-19 by sex differences and age. Womens Health. 2021;17:17455065211022262. 10.1177/17455065211022262 PMC8188967 34096383

[CR26] Coles MJ, Masood M, Crowley MM, Hudgi A, Okereke C, Klein J. It ain’t over ’til it’s over: SARS CoV-2 and post-infectious gastrointestinal dysmotility. Dig Dis Sci. 2022;67(12):5407. 35357608 10.1007/s10620-022-07480-1 PMC8968095

[CR27] Cui G-Y, Rao B-C, Zeng Z-H, Wang X-M, Ren T, Wang H-Y, et al. Characterization of oral and gut microbiome and plasma metabolomics in COVID-19 patients after 1-year follow-up. Mil Med Res. 2022;9(1):32. 35715833 10.1186/s40779-022-00387-y PMC9204369

[CR28] Daitch V, Yelin D, Awwad M, Guaraldi G, Milić J, Mussini C, et al. Characteristics of long-COVID among older adults: a cross-sectional study. Int J Infect Dis. 2022;125:287–93. 36191820 10.1016/j.ijid.2022.09.035

[CR29] Dashraath P, Wong JLJ, Lim MXK, Lim LM, Li S, Biswas A, et al. Coronavirus disease 2019 (COVID-19) pandemic and pregnancy. Am J Obstetr Gynecol. 2020;222(6):521. 10.1016/j.ajog.2020.03.021 PMC7270569 32217113

[CR30] Davani-Davari D, Negahdaripour M, Karimzadeh I, Seifan M, Mohkam M, Masoumi SJ, et al. Prebiotics: definition, types, sources, mechanisms, and clinical applications. Foods. 2019;8(3):92. 30857316 10.3390/foods8030092 PMC6463098

[CR31] de Oliveira AP, Lopes ALF, Pacheco G, de Nolêto IR, Nicolau LAD, Medeiros JV. Premises among SARS-CoV-2, dysbiosis and diarrhea: walking through the ACE2/mTOR/autophagy route. Med Hypotheses. 2020;144: 110243. 33254549 10.1016/j.mehy.2020.110243 PMC7467124

[CR32] Desgranges F, Tadini E, Munting A, Regina J, Filippidis P, Viala B, et al. Post-COVID-19 syndrome in outpatients: a cohort study. J Gen Intern Med. 2022;37(8):1943. 35319081 10.1007/s11606-021-07242-1 PMC8939498

[CR33] Devaux CA, Lagier JC, Raoult D. New insights into the physiopathology of COVID-19: SARS-CoV-2-associated gastrointestinal illness. Front Med. 2021;8:640073. 10.3389/fmed.2021.640073 PMC7930624 33681266

[CR34] di Filippo L, Frara S, Nannipieri F, Cotellessa A, Locatelli M, Rovere Querini P, et al. Low vitamin D levels are associated with long COVID syndrome in COVID-19 survivors. J Clin Endocrinol Metab. 2023. 10.1530/endoabs.90.EP150. 37051747 10.1210/clinem/dgad207 PMC10505553

[CR35] Dieterich W, Schink M, Zopf Y. Microbiota in the gastrointestinal tract. Med Sci. 2018;6(4):116. 10.3390/medsci6040116 PMC6313343 30558253

[CR36] Dominguez-Bello MG, Costello EK, Contreras M, Magris M, Hidalgo G, Fierer N, et al. Delivery mode shapes the acquisition and structure of the initial microbiota across multiple body habitats in newborns. Proc Natl Acad Sci USA. 2010;107(26):11971–5. 20566857 10.1073/pnas.1002601107 PMC2900693

[CR37] Drossman DA, Hasler WL. Rome IV—functional GI disorders: disorders of gut-brain interaction. Gastroenterology. 2016;150(6):1257–61. 27147121 10.1053/j.gastro.2016.03.035

[CR38] Effenberger M, Grabherr F, Mayr L, Schwaerzler J, Nairz M, Seifert M, et al. Faecal calprotectin indicates intestinal inflammation in COVID-19. Gut. 2020;69(8):1543–4. 32312790 10.1136/gutjnl-2020-321388 PMC7211078

[CR39] Entrenas Castillo M, Entrenas Costa LM, Vaquero Barrios JM, Alcalá Díaz JF, López Miranda J, Bouillon R, et al. Effect of calcifediol treatment and best available therapy versus best available therapy on intensive care unit admission and mortality among patients hospitalized for COVID-19: a pilot randomized clinical study. J Steroid Biochem Mol Biol. 2020;203:105751. 32871238 10.1016/j.jsbmb.2020.105751 PMC7456194

[CR40] Fanelli M, Petrone V, Buonifacio M, Delibato E, Balestrieri E, Grelli S, et al. Multidistrict host-pathogen interaction during COVID-19 and the development post-infection chronic inflammation. Pathogens. 2022;11(10):1198. 36297256 10.3390/pathogens11101198 PMC9607297

[CR41] Fernández-de-las-Peñas C, Florencio LL, Gómez-Mayordomo V, Cuadrado ML, Palacios-Ceña D, Raveendran AV. Proposed integrative model for post-COVID symptoms. Diabetes Metab Syndr Clin Res Rev. 2021;15(4):102159. 10.1016/j.dsx.2021.05.032 PMC8168334 34186367

[CR42] Fernández-De-las-Peñas C, Palacios-Ceña D, Gómez-Mayordomo V, Cuadrado ML, Florencio LL. Defining post-COVID symptoms (post-acute COVID, long COVID, persistent post-COVID): an integrative classification. Int J Environ Res Public Health. 2021;18(5):2621. 33807869 10.3390/ijerph18052621 PMC7967389

[CR43] Forsgård RA, Rode J, Lobenius-Palmér K, Kamm A, Patil S, Tacken MGJ, et al. *Limosilactobacillus**reuteri* DSM 17938 supplementation and SARS-CoV-2 specific antibody response in healthy adults: a randomized, triple-blinded, placebo-controlled trial. Gut Microbes. 2023;15(1):2229938. 37401761 10.1080/19490976.2023.2229938 PMC10321188

[CR44] Frank DN, St Amand AL, Feldman RA, Boedeker EC, Harpaz N, Pace NR. Molecular-phylogenetic characterization of microbial community imbalances in human inflammatory bowel diseases. Proc Natl Acad Sci USA. 2007;104(34):13780–5. 17699621 10.1073/pnas.0706625104 PMC1959459

[CR45] Freimer D, Yang TT, Ho TC, Tymofiyeva O, Leung C. The gut microbiota, HPA axis, and brain in adolescent-onset depression: probiotics as a novel treatment. Brain Behav Immun Health. 2022;26:100541. 36536630 10.1016/j.bbih.2022.100541 PMC9758412

[CR46] Gaebler C, Wang Z, Lorenzi JCC, Muecksch F, Finkin S, Tokuyama M, et al. Evolution of antibody immunity to SARS-CoV-2. Nature. 2021;591(7851):639–44. 33461210 10.1038/s41586-021-03207-w PMC8221082

[CR47] Galán M, Vigón L, Fuertes D, Murciano-Antón MA, Casado-Fernández G, Domínguez-Mateos S, et al. Persistent overactive cytotoxic immune response in a Spanish cohort of individuals with long-COVID: identification of diagnostic biomarkers. Front Immunol. 2022;13:1. 10.3389/fimmu.2022.848886 PMC8990790 35401523

[CR48] Gao F, Guo R, Ma Q, Li Y, Wang W, Fan Y, et al. Stressful events induce long-term gut microbiota dysbiosis and associated post-traumatic stress symptoms in healthcare workers fighting against COVID-19. J Affect Disord. 2022;303:187–95. 35157946 10.1016/j.jad.2022.02.024 PMC8837476

[CR49] Gareau MG, Barrett KE. Coronavirus disease (COVID-19) and digestive system: role of the microbiota-gut-brain axis in postacute COVID syndrome. Am J Physiol Gastrointest Liver Physiol. 2023;324(4):G322. 36880667 10.1152/ajpgi.00293.2022 PMC10042594

[CR50] Ghosh TS, Rampelli S, Jeffery IB, Santoro A, Neto M, Capri M, et al. Original research: Mediterranean diet intervention alters the gut microbiome in older people reducing frailty and improving health status: the NU-AGE 1-year dietary intervention across five European countries. Gut. 2020;69(7):1218. 32066625 10.1136/gutjnl-2019-319654 PMC7306987

[CR51] Gold JE, Okyay RA, Licht WE, Hurley DJ. Investigation of long COVID prevalence and its relationship to Epstein-barr virus reactivation. Pathogens. 2021;10(6):763. 34204243 10.3390/pathogens10060763 PMC8233978

[CR52] Gou W, Fu Y, Yue L, Chen G-D, Cai X, Shuai M, et al. Gut microbiota, inflammation, and molecular signatures of host response to infection. J Genetics Genomics. 2021;48(9):792–802. 34257044 10.1016/j.jgg.2021.04.002

[CR53] Groff D, Sun A, Ssentongo AE, Ba DM, Parsons N, Poudel GR, et al. Short-term and long-term rates of postacute sequelae of SARS-CoV-2 infection: a systematic review. JAMA Netw Open. 2021;4(10): e2128568. 34643720 10.1001/jamanetworkopen.2021.28568 PMC8515212

[CR54] Günther C, Rothhammer V, Karow M, Neurath M, Winner B. The gut-brain axis in inflammatory bowel disease—current and future perspectives. Int J Mol Sci. 2021;22(16):22. 10.3390/ijms22168870 PMC8396333 34445575

[CR55] Hadjadj J, Yatim N, Barnabei L, Corneau A, Boussier J, Smith N, et al. Impaired type I interferon activity and inflammatory responses in severe COVID-19 patients. Science (New York, Ny). 2020;369(6504):718. 10.1126/science.abc6027 PMC7402632 32661059

[CR56] Hallmann E, Sikora D, Poniedziałek B, Szymański K, Kondratiuk K, Żurawski J, et al. IgG autoantibodies against ACE2 in SARS-CoV-2 infected patients. J Med Virol. 2023;95(1): e28273. 36324055 10.1002/jmv.28273 PMC9877908

[CR57] Hany M, Sheta E, Talha A, Anwar M, Selima M, Gaballah M, et al. Incidence of persistent SARS-CoV-2 gut infection in patients with a history of COVID-19: Insights from endoscopic examination. Endosc Int Open. 2024;12(1):E11-e22. 38188925 10.1055/a-2180-9872 PMC10769582

[CR58] Hashimoto K. Detrimental effects of COVID-19 in the brain and therapeutic options for long COVID: the role of Epstein-Barr virus and the gut–brain axis. Mol Psychiatry. 2023;2023:1–9. 10.1038/s41380-023-02161-5 PMC11041741 37402856

[CR59] Haunhorst S, Bloch W, Javelle F, Krüger K, Baumgart S, Drube S, et al. A scoping review of regulatory T cell dynamics in convalescent COVID-19 patients—indications for their potential involvement in the development of Long COVID? Front Immunol. 2022;13:1070994. 36582234 10.3389/fimmu.2022.1070994 PMC9792979

[CR60] Hill C, Guarner F, Reid G, Gibson GR, Merenstein DJ, Pot B, et al. The international scientific association for probiotics and prebiotics consensus statement on the scope and appropriate use of the term probiotic. Nat Rev Gastroenterol Hepatol. 2014;11(8):506–14. 24912386 10.1038/nrgastro.2014.66

[CR61] Hilpert K, Mikut R. Is there a connection between gut microbiome dysbiosis occurring in COVID-19 patients and post-COVID-19 symptoms? Front Microbiol. 2021;12:732838. 34603261 10.3389/fmicb.2021.732838 PMC8485028

[CR62] Hotchkiss AT, Renye JA, White AK, Nunez A, Guron GKP, Chau H, et al. Cranberry arabino-xyloglucan and pectic oligosaccharides induce lactobacillus growth and short-chain fatty acid production. Microorganisms. 2022;10(7):1346. 35889065 10.3390/microorganisms10071346 PMC9319371

[CR63] Ho-Yen DO. The epidemiology of post viral fatigue syndrome. Scott Med J. 1988;33(6):368–9. 2854300 10.1177/003693308803300607

[CR64] Hu J, Zhang L, Lin W, Tang W, Chan FKL, Ng SC. Review article: probiotics, prebiotics and dietary approaches during COVID-19 pandemic. Trends Food Sci Technol. 2021;108:187. 33519087 10.1016/j.tifs.2020.12.009 PMC7833886

[CR65] Huang I, Lim MA, Pranata R. Diabetes mellitus is associated with increased mortality and severity of disease in COVID-19 pneumonia—a systematic review, meta-analysis, and meta-regression. Diabetes Metab Syndr. 2020;14(4):395–403. 32334395 10.1016/j.dsx.2020.04.018 PMC7162793

[CR66] Iqbal P, Ata F, Chaudhry H, Muthanna B, Waqas Younas H, Munamm SA, et al. Post-COVID-19-associated multiorgan complications or “long COVID” with literature review and management strategy discussion: a meta-analysis. Health Sci Rep. 2023;6(4):1211. 10.1002/hsr2.1211 PMC10103688 37064319

[CR67] Kaushik P, Kumari M, Singh NK, Suri A. The role of gut microbiota in etiopathogenesis of long COVID syndrome. Horm Mol Biol Clin Invest. 2022;44(2):113–4. 10.1515/hmbci-2022-0079 36317311

[CR68] Klann EM, Dissanayake U, Gurrala A, Farrer M, Shukla AW, Ramirez-Zamora A, et al. The gut-brain axis and its relation to Parkinson’s disease: a review. Front Aging Neurosci. 2021;13: 782082. 35069178 10.3389/fnagi.2021.782082 PMC8776990

[CR69] Klein J, Wood J, Jaycox J, Lu P, Dhodapkar RM, Gehlhausen JR, et al. Distinguishing features of Long COVID identified through immune profiling. medRxiv. 2022. 10.1038/s41586-023-06651-y. 37748514 10.1038/s41586-023-06651-y PMC10620090

[CR70] Koenig JE, Spor A, Scalfone N, Fricker AD, Stombaugh J, Knight R, et al. Succession of microbial consortia in the developing infant gut microbiome. Proc Natl Acad Sci USA. 2011;108(Suppl 1):4578–85. 20668239 10.1073/pnas.1000081107 PMC3063592

[CR71] Kurian SJ, Unnikrishnan MK, Miraj SS, Bagchi D, Banerjee M, Reddy BS, et al. Probiotics in prevention and treatment of COVID-19: current perspective and future prospects. Arch Med Res. 2021;52(6):582. 33785208 10.1016/j.arcmed.2021.03.002 PMC7972717

[CR72] Lavery AM, Preston LE, Ko JY, Chevinsky JR, DeSisto CL, Pennington AF, et al. Characteristics of hospitalized COVID-19 patients discharged and experiencing same-hospital readmission—United States, March–August 2020. Morb Mortal Wkly Rep. 2020;69(45):1695. 10.15585/mmwr.mm6945e2 PMC7660660 33180754

[CR73] Lechien JR, Chiesa-Estomba CM, Place S, Van Laethem Y, Cabaraux P, Mat Q, et al. Clinical and epidemiological characteristics of 1420 European patients with mild-to-moderate coronavirus disease 2019. J Intern Med. 2020;288(3):335. 32352202 10.1111/joim.13089 PMC7267446

[CR74] Lee JY, Tsolis RM, Bäumler AJ. The microbiome and gut homeostasis. Science. 2022;377(6601):eabp9960. 35771903 10.1126/science.abp9960

[CR75] Lippi G, Sanchis-Gomar F, Henry BM. COVID-19 and its long-term sequelae: what do we know in 2023? Pol Arch Intern Med. 2023;133(4):16402. 36626183 10.20452/pamw.16402

[CR76] Liu YW, Liu WH, Wu CC, Juan YC, Wu YC, Tsai HP, et al. Psychotropic effects of *Lactobacillus**plantarum* PS128 in early life-stressed and naïve adult mice. Brain Res. 2016;1631:1–12. 26620542 10.1016/j.brainres.2015.11.018

[CR77] Liu Q, Su Q, Zhang F, Tun HM, Mak JWY, Lui GCY, et al. Multi-kingdom gut microbiota analyses define COVID-19 severity and post-acute COVID-19 syndrome. Nat Commun. 2022a;13(1):1–11. 36357381 10.1038/s41467-022-34535-8 PMC9648868

[CR78] Liu Q, Mak JWY, Su Q, Yeoh YK, Lui GCY, Ng SSS, et al. Original research: gut microbiota dynamics in a prospective cohort of patients with post-acute COVID-19 syndrome. Gut. 2022b. 10.1136/gutjnl-2021-325989. 35082169 10.1136/gutjnl-2021-325989

[CR79] Logue JK, Franko NM, McCulloch DJ, McConald D, Magedson A, Wolf CR, et al. Sequelae in adults at 6 months after COVID-19 infection. JAMA Netw Open. 2021;4(2): e210830. 33606031 10.1001/jamanetworkopen.2021.0830 PMC7896197

[CR80] Łoniewski I, Skonieczna-Żydecka K, Sołek-Pastuszka J, Marlicz W. Probiotics in the management of mental and gastrointestinal post-COVID symptomes. J Clin Med. 2022;11(17):5155. 36079082 10.3390/jcm11175155 PMC9457065

[CR81] Ma Y, Deng J, Liu Q, Du M, Liu M, Liu J. Long-term consequences of COVID-19 at 6 months and above: a systematic review and meta-analysis. Int J Environ Res Public Health. 2022;19(11):6865. 35682448 10.3390/ijerph19116865 PMC9180091

[CR82] Mahmud R, Rahman MM, Rassel MA, Monayem FB, Sayeed SKJB, Islam MS, et al. Post-COVID-19 syndrome among symptomatic COVID-19 patients: a prospective cohort study in a tertiary care center of Bangladesh. PLoS ONE. 2021;16(4): e0249644. 33831043 10.1371/journal.pone.0249644 PMC8031743

[CR83] Markov PV, Ghafari M, Beer M, Lythgoe K, Simmonds P, Stilianakis NI, et al. The evolution of SARS-CoV-2. Nat Rev Microbiol. 2023;21(6):361–79. 37020110 10.1038/s41579-023-00878-2

[CR84] Mayer EA. Gut feelings: the emerging biology of gut–brain communication. Nat Rev Neurosci. 2011;12(8):453–66. 21750565 10.1038/nrn3071 PMC3845678

[CR85] McLoughlin RF, Berthon BS, Jensen ME, Baines KJ, Wood LG. Short-chain fatty acids, prebiotics, synbiotics, and systemic inflammation: a systematic review and meta-analysis. Am J Clin Nutr. 2017;106(3):930–45. 28793992 10.3945/ajcn.117.156265

[CR86] Megur A, Baltriukienė D, Bukelskienė V, Burokas A. The microbiota-gut-brain axis and Alzheimer’s disease: neuroinflammation is to blame? Nutrients. 2020;13(1):37. 33374235 10.3390/nu13010037 PMC7824474

[CR87] Merino J, Joshi AD, Nguyen LH, Leeming ER, Mazidi M, Drew DA, et al. Diet quality and risk and severity of COVID-19: a prospective cohort study. Gut. 2021;70(11):2096. 34489306 10.1136/gutjnl-2021-325353 PMC8500931

[CR88] Mukhtar K, Nawaz H, Abid S. Functional gastrointestinal disorders and gut-brain axis: what does the future hold? World J Gastroenterol. 2019;25(5):552. 30774271 10.3748/wjg.v25.i5.552 PMC6371005

[CR89] Munblit D, Nicholson T, Akrami A, Apfelbacher C, Chen J, De Groote W, et al. A core outcome set for post-COVID-19 condition in adults for use in clinical practice and research: an international Delphi consensus study. Lancet Respir Med. 2022;10(7):715. 35714658 10.1016/S2213-2600(22)00169-2 PMC9197249

[CR90] Munipalli B, Seim L, Dawson NL, Knight D, Dabrh AMA. Post-acute sequelae of COVID-19 (PASC): a meta-narrative review of pathophysiology, prevalence, and management. Sn comprehensive. Clin Med. 2022;4(1):90. 10.1007/s42399-022-01167-4 PMC8977184 35402784

[CR91] Muri J, Cecchinato V, Cavalli A, Shanbhag AA, Matkovic M, Biggiogero M, et al. Autoantibodies against chemokines post-SARS-CoV-2 infection correlate with disease course. Nat Immunol. 2023;24(4):604–11. 36879067 10.1038/s41590-023-01445-w PMC10063443

[CR92] Nalbandian A, Sehgal K, Gupta A, Madhavan MV, McGroder C, Stevens JS, et al. Post-acute COVID-19 syndrome. Nat Med. 2021;27(4):601. 33753937 10.1038/s41591-021-01283-z PMC8893149

[CR93] Natarajan A, Zlitni S, Brooks EF, Vance SE, Dahlen A, Hedlin H, et al. Gastrointestinal symptoms and fecal shedding of SARS-CoV-2 RNA suggest prolonged gastrointestinal infection. Med (New York, Ny). 2022;3(6):371. 10.1016/j.medj.2022.04.001 PMC9005383 35434682

[CR94] National centre for health statistics: nearly one in five American adults who have had COVID-19 still have “Long COVID”. 2022.

[CR95] Neris Almeida Viana S, da Reis Santos Pereria T, de Carvalho Alves J, de Tianeze Castro C, da Santana Silva LC, Henrique Sousa Pinheiro L, et al. Benefits of probiotic use on COVID-19: a systematic review and meta-analysis. Crit Rev Food Sci Nutr. 2022. 10.1080/10408398.2022.2128713. 36178362 10.1080/10408398.2022.2128713

[CR96] NICE. COVID-19 rapid guideline: managing the long-term effects of COVID-19. 2020. 33555768

[CR97] Ning Q, Wu D, Wang X, Xi D, Chen T, Chen G, et al. The mechanism underlying extrapulmonary complications of the coronavirus disease 2019 and its therapeutic implication. Signal Transd Target Ther. 2022;7(1):1–33. 10.1038/s41392-022-00907-1 PMC8863906 35197452

[CR98] Olaimat AN, Aolymat I, Al-Holy M, Ayyash M, Abu Ghoush M, Al-Nabulsi AA, et al. The potential application of probiotics and prebiotics for the prevention and treatment of COVID-19. NPJ Sci Food. 2020;4(1):1–7. 33083549 10.1038/s41538-020-00078-9 PMC7536434

[CR99] Olofsson PS, Katz DA, Rosas-Ballina M, Levine YA, Ochani M, Valdés-Ferrer SI, et al. α7 nicotinic acetylcholine receptor (α7nAChR) expression in bone marrow-derived non–T cells is required for the inflammatory reflex. Mol Med. 2012;18(1):539. 22183893 10.2119/molmed.2011.00405 PMC3356417

[CR100] Parker A, Fonseca S, Carding SR. Gut microbes and metabolites as modulators of blood-brain barrier integrity and brain health. Gut Microbes. 2020;11(2):135. 31368397 10.1080/19490976.2019.1638722 PMC7053956

[CR101] Pasini E, Corsetti G, Romano C, Scarabelli TM, Chen-Scarabelli C, Saravolatz L, et al. Serum metabolic profile in patients with long-COVID (PASC) syndrome: clinical implications. Front Med. 2021;8:714426. 10.3389/fmed.2021.714426 PMC8339407 34368201

[CR102] Patterson BK, Guevara-Coto J, Yogendra R, Francisco EB, Long E, Pise A, et al. Immune-based prediction of COVID-19 severity and chronicity decoded using machine learning. Front Immunol. 2021;12:700782. 34262570 10.3389/fimmu.2021.700782 PMC8273732

[CR103] Perlot T, Penninger JM. ACE2-from the renin–angiotensin system to gut microbiota and malnutrition. Microb Infect. 2013;15(13):866. 10.1016/j.micinf.2013.08.003 PMC7110844 23962453

[CR104] Perumal R, Shunmugam L, Naidoo K, Abdool Karim SS, Wilkins D, Garzino-Demo A, et al. Long COVID: a review and proposed visualization of the complexity of long COVID. Front Immunol. 2023;14:1117464. 37153597 10.3389/fimmu.2023.1117464 PMC10157068

[CR105] Phetsouphanh C, Darley DR, Wilson DB, Howe A, Munier CML, Patel SK, et al. Immunological dysfunction persists for 8 months following initial mild-to-moderate SARS-CoV-2 infection. Nat Immunol. 2022;23(2):210–6. 35027728 10.1038/s41590-021-01113-x

[CR106] Post COVID-19 condition (long COVID). 2024.

[CR107] Prasad R, Patton MJ, Floyd JL, Fortmann S, DuPont M, Harbour A, et al. Plasma microbiome in COVID-19 subjects: an indicator of gut barrier defects and dysbiosis. Int J Mol Sci. 2022;23(16):9141. 36012406 10.3390/ijms23169141 PMC9409329

[CR108] Premraj L, Kannapadi NV, Briggs J, Seal SM, Battaglini D, Fanning J, et al. Mid and long-term neurological and neuropsychiatric manifestations of post-COVID-19 syndrome: a meta-analysis. J Neurol Sci. 2022;434:120162. 35121209 10.1016/j.jns.2022.120162 PMC8798975

[CR109] Qamar MA, Martins RS, Dhillon RA, Tharwani A, Irfan O, Suriya QF, et al. Residual symptoms and the quality of life in individuals recovered from COVID-19 infection: a survey from Pakistan. Ann Med Surg. 2022;75:103361. 10.1016/j.amsu.2022.103361 PMC8832846 35186286

[CR110] Qi F, Qian S, Zhang S, Zhang Z. Single cell RNA sequencing of 13 human tissues identify cell types and receptors of human coronaviruses. Biochem Biophys Res Commun. 2020;526(1):135–40. 32199615 10.1016/j.bbrc.2020.03.044 PMC7156119

[CR111] Queiroz MA, Neves PF, Lima SS, Lopes JdC, Torres MK, Vallinoto IM, et al. Cytokine profiles associated with acute COVID-19 and long COVID-19 syndrome. Front Cell Infect Microbiol. 2022;12:1. 10.3389/fcimb.2022.922422 PMC9279918 35846757

[CR112] Rajilić-Stojanović M, de Vos WM. The first 1000 cultured species of the human gastrointestinal microbiota. Fems Microbiol Rev. 2014;38(5):996. 24861948 10.1111/1574-6976.12075 PMC4262072

[CR113] Ramakrishnan RK, Kashour T, Hamid Q, Halwani R, Tleyjeh IM. Unraveling the mystery surrounding post-acute sequelae of COVID-19. Front Immunol. 2021;12:686029. 34276671 10.3389/fimmu.2021.686029 PMC8278217

[CR114] Rathi A, Jadhav SB, Shah N. A randomized controlled trial of the efficacy of systemic enzymes and probiotics in the resolution of post-COVID fatigue. Medicines. 2021;8(9):47. 34564089 10.3390/medicines8090047 PMC8472462

[CR115] Raveendran AV, Misra A. Post COVID-19 syndrome (“Long COVID”) and diabetes: challenges in diagnosis and management. Diabetes Metab Syndr. 2021;15(5):102235. 34384972 10.1016/j.dsx.2021.102235 PMC8317446

[CR116] Ronan V, Yeasin R, Claud EC. Childhood development and the microbiome-the intestinal microbiota in maintenance of health and development of disease during childhood development. Gastroenterology. 2021;160(2):495–506. 33307032 10.1053/j.gastro.2020.08.065 PMC8714606

[CR117] Rubas NC, Peres R, Kunihiro BP, Allan NP, Phankitnirundorn K, Wells RK, et al. HMGB1 mediates microbiome-immune axis dysregulation underlying reduced neutralization capacity in obesity-related post-acute sequelae of SARS-CoV-2. Sci Rep. 2024;14(1):355. 38172612 10.1038/s41598-023-50027-1 PMC10764757

[CR118] Russo F, Linsalata M, Clemente C, Chiloiro M, Orlando A, Marconi E, et al. Inulin-enriched pasta improves intestinal permeability and modifies the circulating levels of zonulin and glucagon-like peptide 2 in healthy young volunteers. Nutr Res. 2012;32(12):940–6. 23244539 10.1016/j.nutres.2012.09.010

[CR119] Sălcudean A, Nan AG, Bodo CR, Cosma MC, Strete EG, Lica MM. Association between childhood onset inflammatory bowel disease and psychiatric comorbidities in adulthood. Diagnostics. 2023;13(11):1868. 37296719 10.3390/diagnostics13111868 PMC10252471

[CR120] Scherer PE, Kirwan JP, Rosen CJ. Post-acute sequelae of COVID-19: a metabolic perspective. Elife. 2022;11:78200. 10.7554/eLife.78200 PMC8942467 35318939

[CR121] Sekirov I, Russell SL, Antunes LCM, Finlay BB. Gut Microbiota in health and disease. Physiol Rev. 2010;90(3):859–904. 20664075 10.1152/physrev.00045.2009

[CR122] Shenoy S. Gut microbiome, Vitamin D, ACE2 interactions are critical factors in immune-senescence and inflammaging: key for vaccine response and severity of COVID-19 infection. Inflamm Res. 2022;71(1):13. 34738147 10.1007/s00011-021-01510-w PMC8568567

[CR123] Shuwa HA, Shaw TN, Knight SB, Wemyss K, McClure FA, Pearmain L, et al. Alterations in T and B cell function persist in convalescent COVID-19 patients. Med (New York, Ny). 2021;2(6):720. 10.1016/j.medj.2021.03.013 PMC8011689 33821250

[CR124] Sigfrid L, Drake TM, Pauley E, Jesudason EC, Olliaro P, Lim WS, et al. Long COVID in adults discharged from UK hospitals after COVID-19: a prospective, multicentre cohort study using the ISARIC WHO clinical characterisation protocol. Lancet Reg Health. 2021;8:100186. 10.1016/j.lanepe.2021.100186 PMC8343377 34386785

[CR125] Simadibrata DM, Lesmana E, Gunawan J, Quigley EM, Simadibrata M. A systematic review of gut microbiota profile in COVID-19 patients and among those who have recovered from COVID-19. J Dig Dis. 2023;24(4):244–61. 37265376 10.1111/1751-2980.13195

[CR126] Smyth N, Alwan NA, Band R, Chaudhry A, Chew-Graham CA, Gopal D, et al. Exploring the lived experience of long covid in black and minority ethnic groups in the UK: protocol for qualitative interviews and art-based methods. PLoS ONE. 2022;17(10): e0275166. 36191007 10.1371/journal.pone.0275166 PMC9529129

[CR127] Snelson M, de Pasquale C, Ekinci EI, Coughlan MT. Gut microbiome, prebiotics, intestinal permeability and diabetes complications. Best Pract Res Clin Endocrinol Metab. 2021;35(3):101507. 33642218 10.1016/j.beem.2021.101507

[CR128] Sokol H, Pigneur B, Watterlot L, Lakhdari O, Bermúdez-Humarán LG, Gratadoux JJ, et al. Faecalibacterium prausnitzii is an anti-inflammatory commensal bacterium identified by gut microbiota analysis of Crohn disease patients. Proc Natl Acad Sci USA. 2008;105(43):16731–6. 18936492 10.1073/pnas.0804812105 PMC2575488

[CR129] Soriano JB, Murthy S, Marshall JC, Relan P, Diaz JV. A clinical case definition of post-COVID-19 condition by a Delphi consensus. Lancet Infect Dis. 2022;22(4): e102. 34951953 10.1016/S1473-3099(21)00703-9 PMC8691845

[CR130] Stewart CJ, Ajami NJ, O’Brien JL, Hutchinson DS, Smith DP, Wong MC, et al. Temporal development of the gut microbiome in early childhood from the TEDDY study. Nature. 2018;562(7728):583–8. 30356187 10.1038/s41586-018-0617-x PMC6415775

[CR131] Strandwitz P. Neurotransmitter modulation by the gut microbiota. Brain Res. 2018;1693(Pt B):128. 29903615 10.1016/j.brainres.2018.03.015 PMC6005194

[CR132] Su Q, Liu Q, Zhang L, Xu Z, Liu C, Lu W, et al. Antibiotics and probiotics impact gut antimicrobial resistance gene reservoir in COVID-19 patients. Gut Microbes. 2022;14(1):2128603. 36201636 10.1080/19490976.2022.2128603 PMC9543044

[CR133] Su Y, Yuan D, Chen DG, Ng RH, Wang K, Choi J, et al. Multiple early factors anticipate post-acute COVID-19 sequelae. Cell. 2022a;185(5):881. 35216672 10.1016/j.cell.2022.01.014 PMC8786632

[CR134] Subramanian S, Huq S, Yatsunenko T, Haque R, Mahfuz M, Alam MA, et al. Persistent gut microbiota immaturity in malnourished Bangladeshi children. Nature. 2014;510(7505):417–21. 24896187 10.1038/nature13421 PMC4189846

[CR135] Subramanian A, Nirantharakumar K, Hughes S, Myles P, Williams T, Gokhale KM, et al. Symptoms and risk factors for long COVID in non-hospitalized adults. Nat Med. 2022;28(8):1706. 35879616 10.1038/s41591-022-01909-w PMC9388369

[CR136] Szabo S, Zayachkivska O, Hussain A, Muller V. What is really ‘long COVID’? Inflammopharmacology. 2023;31(2):551. 36964860 10.1007/s10787-023-01194-0 PMC10039447

[CR137] Takahashi T, Ellingson MK, Wong P, Israelow B, Lucas C, Klein J, et al. Sex differences in immune responses that underlie COVID-19 disease outcomes. Nature. 2020;588(7837):315. 32846427 10.1038/s41586-020-2700-3 PMC7725931

[CR138] Thaweethai T, Jolley SE, Karlson EW, Levitan EB, Levy B, McComsey GA, et al. Development of a definition of postacute sequelae of SARS-CoV-2 infection. JAMA. 2023. 10.1001/jama.2023.15712. 37278994 10.1001/jama.2023.8823 PMC10214179

[CR140] Thursby E, Juge N. Introduction to the human gut microbiota. Biochem J. 2017;474(11):1823. 28512250 10.1042/BCJ20160510 PMC5433529

[CR141] Tian Y, Sun KY, Meng TQ, Ye Z, Guo SM, Li ZM, et al. Gut microbiota may not be fully restored in recovered COVID-19 patients after 3-month recovery. Front Nutr. 2021;8:638825. 34055851 10.3389/fnut.2021.638825 PMC8155354

[CR142] Tran SMS, Hasan MM. The role of gut bacterial metabolites in brain development, aging and disease. Nutrients. 2021;13(3):1–41. 10.3390/nu13030732 PMC7996516 33669008

[CR143] Tsigalou C, Konstantinidis T, Paraschaki A, Stavropoulou E, Voidarou C, Bezirtzoglou E. Mediterranean diet as a tool to combat inflammation and chronic diseases. An overview. Biomedicines. 2020;8(7):201. 32650619 10.3390/biomedicines8070201 PMC7400632

[CR144] Tsilingiris D, Vallianou NG, Karampela I, Christodoulatos GS, Papavasileiou G, Petropoulou D, et al. Laboratory findings and biomarkers in long COVID: what do we know so far? Insights into epidemiology, pathogenesis, therapeutic perspectives and challenges. Int J Mol Sci. 2023;24(13):10458. 37445634 10.3390/ijms241310458 PMC10341908

[CR145] Umesh A, Pranay K, Pandey RC, Gupta MK. Evidence mapping and review of long-COVID and its underlying pathophysiological mechanism. Infection. 2022;50(5):1053. 35489015 10.1007/s15010-022-01835-6 PMC9055372

[CR146] Vaga S, Lee S, Ji B, Andreasson A, Talley NJ, Agréus L, et al. Compositional and functional differences of the mucosal microbiota along the intestine of healthy individuals. Sci Rep. 2020;10(1):1–12. 32917913 10.1038/s41598-020-71939-2 PMC7486370

[CR147] Vakili K, Fathi M, Yaghoobpoor S, Sayehmiri F, Nazerian Y, Nazerian A, et al. The contribution of gut-brain axis to development of neurological symptoms in COVID-19 recovered patients: a hypothesis and review of literature. Front Cell Infect Microbiol. 2022;12:983089. 36619768 10.3389/fcimb.2022.983089 PMC9815719

[CR148] Vangay P, Ward T, Gerber JS, Knights D. Antibiotics, pediatric dysbiosis, and disease. Cell Host Microbe. 2015;17(5):553–64. 25974298 10.1016/j.chom.2015.04.006 PMC5555213

[CR149] Villapol S. Gastrointestinal symptoms associated with COVID-19: impact on the gut microbiome. Transl Res. 2020;226:57. 32827705 10.1016/j.trsl.2020.08.004 PMC7438210

[CR150] Vimercati L, De Maria L, Quarato M, Caputi A, Gesualdo L, Migliore G, et al. Association between long COVID and overweight/obesity. J Clin Med. 2021;10(18):4143. 34575251 10.3390/jcm10184143 PMC8469321

[CR151] Vojdani A, Vojdani E, Saidara E, Maes M. Persistent SARS-CoV-2 infection, EBV, HHV-6 and other factors may contribute to inflammation and autoimmunity in long COVID. Viruses. 2023;15(2):400. 36851614 10.3390/v15020400 PMC9967513

[CR152] Vrettou CS, Mantziou V, Vassiliou AG, Dimopoulou I, Orfanos SE, Kotanidou A. Post-intensive care syndrome in survivors from critical illness including COVID-19 patients: a narrative review. Life. 2022;12(1):107. 35054500 10.3390/life12010107 PMC8778667

[CR153] Wang TT, Nestel FP, Bourdeau V, Nagai Y, Wang Q, Liao J, et al. Cutting edge: 1,25-dihydroxyvitamin D3 is a direct inducer of antimicrobial peptide gene expression. J Immunol (Baltimore, Md: 1950). 2004;173(5):2909–12. 10.4049/jimmunol.173.5.2909 15322146

[CR154] Wang H, Ji Y, Wu G, Sun K, Sun Y, Li W, et al. L-tryptophan activates mammalian target of rapamycin and enhances expression of tight junction proteins in intestinal porcine epithelial cells. J Nutr. 2015;145(6):1156–62. 25878205 10.3945/jn.114.209817

[CR155] Wang B, Zhang L, Wang Y, Dai T, Qin Z, Zhou F, et al. Alterations in microbiota of patients with COVID-19: potential mechanisms and therapeutic interventions. Signal Transduct Target Ther. 2022;7(1):1–15. 35487886 10.1038/s41392-022-00986-0 PMC9052735

[CR156] Webers A, Heneka MT, Gleeson PA. The role of innate immune responses and neuroinflammation in amyloid accumulation and progression of Alzheimer’s disease. Immunol Cell Biol. 2020;98(1):28–41. 31654430 10.1111/imcb.12301

[CR157] Whitaker M, Elliott J, Chadeau-Hyam M, Riley S, Darzi A, Cooke G, et al. Persistent COVID-19 symptoms in a community study of 606,434 people in England. Nat Commun. 2022;13(1):1–10. 35413949 10.1038/s41467-022-29521-z PMC9005552

[CR158] Wu C, Xu Q, Cao Z, Pan D, Zhu Y, Wang S, et al. The volatile and heterogeneous gut microbiota shifts of COVID-19 patients over the course of a probiotics-assisted therapy. Clin Transl Med. 2021;11(12): e643. 34962356 10.1002/ctm2.643 PMC8713143

[CR159] Xu J, Ren Z, Cao K, Li X, Yang J, Luo X, et al. Boosting vaccine-elicited respiratory mucosal and systemic COVID-19 immunity in mice with the oral *Lactobacillus**plantarum*. Front Nutr. 2021;8:789242. 35004816 10.3389/fnut.2021.789242 PMC8733898

[CR160] Yeoh YK, Zuo T, Lui GCY, Zhang F, Liu Q, Li AYL, et al. Original research: gut microbiota composition reflects disease severity and dysfunctional immune responses in patients with COVID-19. Gut. 2021;70(4):698. 33431578 10.1136/gutjnl-2020-323020 PMC7804842

[CR161] Yeoh YK, Zuo T, Lui GC, Zhang F, Liu Q, Li AY, et al. Gut microbiota composition reflects disease severity and dysfunctional immune responses in patients with COVID-19. Gut. 2021;70(4):698–706. 33431578 10.1136/gutjnl-2020-323020 PMC7804842

[CR162] Yin JX, Agbana YL, Sun ZS, Fei SW, Zhao HQ, Zhou XN, et al. Increased interleukin-6 is associated with long COVID-19: a systematic review and meta-analysis. Infect Dis Pov. 2023;12(1):43. 10.1186/s40249-023-01086-z PMC10123579 37095536

[CR163] Yomogida K, Zhu S, Rubino F, Figueroa W, Balanji N, Holman E. Post-acute sequelae of SARS-CoV-2 infection among adults aged ≥18 years—Long Beach, California, April 1–December 10, 2020. Morb Mortal Wkly Rep. 2021;70(37):1274. 10.15585/mmwr.mm7037a2 PMC8445372 34529639

[CR164] Yong SJ, Halim A, Halim M, Liu S, Aljeldah M, Al Shammari BR, et al. Inflammatory and vascular biomarkers in post-COVID-19 syndrome: a systematic review and meta-analysis of over 20 biomarkers. Rev Med Virol. 2023;33(2): e2424. 36708022 10.1002/rmv.2424

[CR165] Yu Y, Zhao F. Microbiota-gut-brain axis in autism spectrum disorder. J Genetics Genomics. 2021;48(9):755–62. 34373221 10.1016/j.jgg.2021.07.001

[CR166] Zafar H, Saier MH. Gut bacteroides species in health and disease. Gut Microbes. 2021;13(1):1–20. 33535896 10.1080/19490976.2020.1848158 PMC7872030

[CR167] Zhan D, Zho Y, Yanling M, Che P, Tan J, Yan B, et al. Gut microbiota dysbiosis correlates with long COVID-19 at one-year after discharge. J Kor Med Sci. 2023;38(15): e120. 10.3346/jkms.2023.38.e120 PMC10111044 37069814

[CR168] Zhang F, Wan Y, Zuo T, Yeoh YK, Liu Q, Zhang L, et al. Prolonged impairment of short-chain fatty acid and l-isoleucine biosynthesis in gut microbiome in patients with COVID-19. Gastroenterology. 2022a;162(2):548. 34687739 10.1053/j.gastro.2021.10.013 PMC8529231

[CR169] Zhang L, Xu Z, Mak JWY, Chow KM, Lui G, Li TCM, et al. Gut microbiota-derived synbiotic formula (SIM01) as a novel adjuvant therapy for COVID-19: an open-label pilot study. J Gastroenterol Hepatol. 2022b;37(5):823. 35170078 10.1111/jgh.15796 PMC9115073

[CR170] Zhang F, Lau RI, Liu Q, Su Q, Chan FKL, Ng SC. Gut microbiota in COVID-19: key microbial changes, potential mechanisms and clinical applications. Nat Rev Gastroenterol Hepatol. 2023;20(5):323–37. 36271144 10.1038/s41575-022-00698-4 PMC9589856

[CR171] Zheng YB, Zeng N, Yuan K, Tian SS, Yang YB, Gao N, et al. Prevalence and risk factor for long COVID in children and adolescents: a meta-analysis and systematic review. J Infect Public Health. 2023;16(5):660. 36931142 10.1016/j.jiph.2023.03.005 PMC9990879

[CR172] Zuo T, Zhang F, Lui GCY, Yeoh YK, Li AYL, Zhan H, et al. Alterations in gut microbiota of patients with COVID-19 during time of hospitalization. Gastroenterology. 2020a;159(3):944-55.e8. 32442562 10.1053/j.gastro.2020.05.048 PMC7237927

